# Generation of somatic mitochondrial DNA-replaced cells for mitochondrial dysfunction treatment

**DOI:** 10.1038/s41598-021-90316-1

**Published:** 2021-05-25

**Authors:** Hideki Maeda, Daisuke Kami, Ryotaro Maeda, Akira Shikuma, Satoshi Gojo

**Affiliations:** 1 Department of Cardiovascular Medicine, Kyoto Prefectural University of Medicine, 465 Kajii cho, Kamigyo ku, Kyoto, 602-8566 Japan; 2 Department of Regenerative Medicine, Kyoto Prefectural University of Medicine, 465 Kajii cho, Kamigyo ku, Kyoto, 602-8566 Japan

**Keywords:** Metabolic engineering, Organelles

## Abstract

Mitochondrial diseases currently have no cure regardless of whether the cause is a nuclear or mitochondrial genome mutation. Mitochondrial dysfunction notably affects a wide range of disorders in aged individuals, including neurodegenerative diseases, cancers, and even senescence. Here, we present a procedure to generate mitochondrial DNA-replaced somatic cells with a combination of a temporal reduction in endogenous mitochondrial DNA and coincubation with exogeneous isolated mitochondria. Heteroplasmy in mitochondrial disease patient-derived fibroblasts in which the mutant genotype was dominant over the wild-type genotype was reversed. Mitochondrial disease patient-derived fibroblasts regained respiratory function and showed lifespan extension. Mitochondrial membranous components were utilized as a vehicle to deliver the genetic materials into endogenous mitochondria-like horizontal genetic transfer in prokaryotes. Mitochondrial DNA-replaced cells could be a resource for transplantation to treat maternal inherited mitochondrial diseases.

## Introduction

Mammalian mitochondria are composed of more than 1,500 proteins and contain a circular genome, mitochondrial DNA (mtDNA), with several copy numbers (CNs) within a matrix encircled by double membranes and the intermembrane space; mtDNA has been reduced to 16.6 kb during evolution through gene transfer to the nucleus^1^. Only 13 mitochondrial proteins are encoded by mtDNA, in addition to 22 tRNAs and 2 rRNAs. Mitochondrial diseases are one of the relatively rare types of inherited metabolic disorders and include two discrete groups that are caused by mutations in mitochondrial or nuclear DNA^2^. Based on symptoms, not on the types of mutations, mitochondrial diseases are subtyped into a number of syndromes, such as mitochondrial myopathy, encephalomyopathy, lactic acidosis, stroke-like symptoms (MELAS), myoclonic epilepsy with ragged red fibers (MERRF), and Leigh syndrome^3^. Because mitochondria perform essential functions in cellular physiology, such as energy generation^4^, innate immunity^5^, Ca kinetics^6^, and apoptosis^7^, mitochondrial dysregulation and defects lead to various clinical manifestations in many organs at any age, depending on the causative mutations, which may emerge de novo sporadically or are inherited from an autosome, the X chromosome, or maternally. Mitochondrial diseases with pathological mutations in mtDNA display a phenotype when the rate of mutated versus healthy mtDNA, termed heteroplasmy^8,9^, surpasses a given critical threshold (typically 60 – 80%) to biochemical disturbances and defective respiration^10^. As the clinical phenotypes of MELAS and MERRF correlate to the heteroplasmy level in muscle but not in blood^11^, there is heterogeneity in the heteroplasmy level between cells even in the same tissue or organ, from organ to organ in the same person^12^. The heterogeneity of mtDNA is divided into intracellular and intercellular heteroplasmy^13^, and a single-cell analysis in mitochondrial biology has just emerged for clinical samples^14,15^. Current knowledge on mitochondrial diseases has not resulted in the precise prediction and prognosis of patients. Although there has been no curative treatment for mitochondrial diseases and only symptomatic therapy has been provided to patients, understanding mechanistic insights into the diseases has led to the development of clinical trials using small molecules as well as cell or gene therapy^16^.

At the beginning of the emergence of eukaryotes, an α-proteobacterium was engulfed by the progenitor, enabling evolution^2^. The capability to take environmental materials up into the cytosol, such as through pinocytosis and especially macropinocytosis, when engulfing large materials is preserved with the current cells and organisms^17,18^. Horizontal transfer of genetic materials is a common characteristic of bacteria such as *E. coli*; however, these phenomena are also observed in mammalian cells^19^. Since mammalian horizontal transfer of mitochondria through a tunneling nanotube was reported in an in vitro experiment^20^, mitochondrial transfer has been demonstrated with various mechanisms, such as nanotubes^21^, extracellular vesicles^22^, and macropinocytosis^23^, as well as by using an artificial device^24^ and a compound^25^. Although the efficiency of mitochondrial transfer did not dominate over the pre-existing mtDNA but provided additive functionality in the host cells, mechanistic insights into mitochondrial transfer with respect to cell entry and endosomal escape have been deepened^26^.

The concept of mitochondrial replacement was reported almost three decades ago; this approach utilizes chemicals toxic to mtDNA and enucleated cells as donors of cytosolic organelles, including mitochondria^27^. Long-term exposure of cells to ethidium bromide under culture conditions gives rise to mtDNA-free cells, termed ρ0. The in vitro enucleation technique^28^ enabled the generation of cells with replaced mitochondria, named cybrid cells, through the fusion of ρ0 cells and enucleated cells. In vivo mitochondrial replacement in model animals was achieved using either embryonic stem (ES) cell cybrids pretreated with rhodamine 6G or mitochondrial injection into oocytes or zygotes^29^. Gene transfer of endonuclease with a mitochondrial transfer signal to cut and degrade mtDNA offered another ρ0 creation method^30^. The mitochondria-targeted endonuclease approach has been utilized to generate a heteroplasmy shift of mitochondrial DNA (mtDNA) in mitochondrial diseases^31^ rather than to form a deletion of mtDNA for cybrid cells, based on the threshold theory of mitochondrial heteroplasmy^10^. Emerging genome editing technology has been extended to the mitochondrial genome to fix a mutation by using mitochondrially targeted Cys2-His2 zinc-finger nucleases (ZFNs)^32^ and transcription activator-like effector nucleases (TALENs)^33^. Clinically, mitochondrial replacement therapy (MRT) in assisted reproductive technologies, which is technically executed by pronuclear transfer, maternal spindle transfer, or polar body transfer, is enthusiastic, especially with respect to ethics^34,35^. Interventions to modulate mtDNA are rising to the level of mitochondrial gene therapy.

In our previous studies, exogenous isolated mitochondria are engulfed by cultured fibroblasts through macropinocytosis^23^, and TAT peptide enhanced the efficacy of engulfment of exogenous mitochondria upon the co-culture setting in primary cardiomyocytes^25^. Because even accelerated engulfment did not exhibit a significant heteroplasmy shift, we focused on the regulation of macropinocytosis to pursue the potential to treat maternal inherited mitochondrial diseases through the heteroplasmy shift. We hypothesized that mtDNA reduction before co-incubation with exogenous mitochondria could lead that exogenous mtDNA takes over mitochondria through macropinocytosis that might be enhanced by the energy crisis with mtDNA reduction. In this study, we demonstrate a protocol to generate somatic mitochondrial DNA-replaced cells (MirCs) and the potential to treat maternal inherited mitochondrial diseases.

## Materials and methods

### Ethical approval and consent to participate

This study was conducted in accordance with Ethical Guidelines for Medical and Health Research Involving Human Subjects in Japan (https://www.mhlw.go.jp/file/06-Seisakujouhou-10600000-Daijinkanboukouseikagakuka/0000080278.pdf), Ethical Guidelines for Human Genome/Gene analysis Research in Japan(https://www.lifescience.mext.go.jp/files/pdf/n2181_02.pdf) and Ministerial Ordinance regarding Recombinant DNA Experiments in Japan (https://www.lifescience.mext.go.jp/files/html/6_27.html) and was accepted by the institutional ethical committee (#ERB-C-1010) and recombinant DNA experiment committee (2019-108) in Kyoto Prefectural University of Medicine. For 7S fibroblasts in this research, the patient with mitochondrial disease have signed a written informed consent form to provide them.

### Plasmid production and transfection

The mitochondrial targeting sequence fused with the 5’-end of DsRed2 (MTS-DsRed2) was digested from the pDsRed2-Mito vector (Clontech Laboratories, Inc., Palo Alto, CA, USA) with restriction enzymes and inserted into the pMXs-puro retroviral vector (Cell Biolabs, Inc. San Diego, CA. USA). The pCAGGS-MTS-*Xba*IR-P2A-PuroR and pCAGGS-MTS-EGFP-P2A-PuroR plasmids were synthesized from by Genewiz (Suzhou, China). The DNA sequence of *Xba*IR referred to AF051092 of NCBI GenBank. These plasmids were transfected into NHDFs and 7SPs by electroporation using the nucleofector kit and nucleofector 2b (Lonza, Walkersvill, MD, USA). Two days later, MTS-*Xba*IR- or MTS-EGFP-transfected cells were purified with 2 µg/ml puromycin treatment for 24 h. The plasmids used to assess the heteroplasmy ratio of mitochondria synthesized the sequence corresponding to the TaqMan probes and inserted them directly by GeneWiz (South Plainfield, NJ, USA).

### Human fibroblast culture

NHDFs were obtained from Lonza (Walkersvill, MD, USA). Leigh syndrome patient-derived skin (7S) fibroblasts were kindly provided by Koinobori Associate Inc., which supports research on mitochondrial diseases under approval from the ethical committees of our institution and Koinobori Associate Inc. The human uterine endometrial gland-derived mesenchymal cell line EPC100 was acquired from the Japanese Collection of Research Bioresources Cell Bank, where it is assigned JCRB1538. NHDFs and EPC100 cells were maintained in high-glucose DMEM (043-30085, Fujifilm Wako Pure Chemical, Osaka, Japan) supplemented with 10% fetal bovine serum and 1% penicillin/streptomycin (Thermo Fisher Scientific Incorporated, Waltham, MA USA). The 7S fibroblasts were maintained in low-glucose DMEM with pyruvate (11885084) supplemented with 10% fetal bovine serum (FBS) and 1% penicillin/streptomycin (Thermo Fisher Scientific incorporated). All cell lines were incubated at 37 °C under 5% CO_2_ and cultured at ~ 80% confluence.

### Cell counting and cell diameter measurement

Cell number and cell viability were measured using the automatic cell counter ADAM (NanoEnTek Inc., Seoul, Korea) according to the manufacturer’s recommendations. Cell diameters were measured using the Scepter 2.0 Cell Counter and Scepter Software Pro (Merck KGaA, Burlington, MA, USA) according to the manufacturer’s recommendations.

### Mitochondrial isolation and transfer to human fibroblasts

Mitochondria were isolated by differential centrifugation as described previously^23^. In brief, the cells were harvested from culture dishes with homogenization buffer [HB; 20 mM HEPES–KOH (pH 7.4), 220 mM mannitol and 70 mM sucrose] containing a protease inhibitor mixture (Sigma-Aldrich, St. Louis, Missouri, USA). The cell pellet was resuspended in HB and incubated on ice for 5 min. The cells were ruptured by 10 strokes of a 27-gauge needle on ice. The homogenate was centrifuged (400 × *g*, 4 °C; 5 min) two times to remove the unbroken cells. The mitochondria were harvested by centrifugation (6000 × *g*, 4 °C; 5 min) and resuspended in HB. The amounts of isolated mitochondria were expressed as the protein concentration using a Bio-Rad protein assay kit (Bio-Rad Laboratories, Incorporated, Richmond, CA, USA). Mitochondrial transfer was conducted by coincubating the isolated mitochondria with the plasmids-transferred cells in 2 ml of standard medium at 37 °C under 5% CO_2_ for 24 h.

### MtDNA digestion and quantitative assay

Total DNA was extracted from cells using NucleoSpin Tissue (Macherey–Nagel, Duren, Germany). The extracted DNA (100 ng) was digested with restriction enzymes (*Xba*IR or *Not*I) at 37 °C for 30 min and subjected to selective amplification by PCR using KOD FX Neo (Toyobo Co., Ltd., Osaka, Japan) under the following conditions: 35 cycles (98 °C for 10 s, 60 °C for 30 s and 68 °C for 30 s) after initial denaturation (94 °C for 2 min). The primers used in this experiment are listed in Supplementary Table S1. Reaction specificity was verified by agarose gel electrophoresis, and DNA bands were visualized using the ChemiDoc XRS + System (Bio-Rad Laboratories, Incorporated).

MtDNA CN was analyzed by quantitative PCR. Quantitative PCR was performed using the extracted DNA (100 ng) as template with Kapa SYBR Fast qPCR Kit Master Mix (2 ×) Universal (Kapa Biosystems Ltd., Wilmington, MA, USA) on a CFX connect real-time system (Bio-Rad Laboratories, Incorporated, Hercules, CA, USA) under the following conditions: 40 cycles of PCR (95 °C for 10 s, 60 °C for 1 min and 72 °C for 30 s) after initial denaturation (95 °C for 2 min).

### MtDNA mutation heteroplasmy analysis

To determine mutation ratios, we designed wild-type and mutant allele-specific TaqMan probes for the TaqMan SNP assay. The extracted DNA (1 ng) was used for quantitative PCR with the TaqMan Universal PCR Master Mix kit (Thermo Fisher Scientific Incorporated) on a CFX connect real-time system (Bio-Rad Laboratories, Incorporated) under the following conditions: 40 cycles of PCR (95 °C for 15 s and 60 °C for 1 min) after initial denaturation (95 °C for 10 min). A calibration curve was created using known CNs of plasmids containing the amplified mtDNA ND3 fragments for either wild-type or mutant sequences. The mtDNA CN was estimated from the content ratio of 12S rRNA on mtDNA and *ACTB* (or *Actb*) on nuclear DNA by delta cycle threshold-based relative quantification.

### RNA isolation, reverse transcription PCR and quantitative PCR

Total RNA from cells was extracted using TRIzol (Thermo Fisher Scientific Incorporated) and a Direct-zol RNA MiniPrep Kit (Zymo Research, Irvine, CA, USA) with DNase I, according to the manufacturer’s recommendations. To perform the qRT-PCR assay, 100 ng of total RNA was reverse-transcribed using the PrimeScript RT Reagent Kit (Takara Bio, Shiga, Japan) and a T100 thermal cycler (Bio-Rad Laboratories, Incorporated). qRT-PCR was performed with Kapa SYBR Fast qPCR Kit Master Mix (2 ×) Universal (Kapa Biosystems Ltd., Wilmington, MA, USA) on a CFX connect real-time system (Bio-Rad Laboratories, incorporated). The relative gene expression levels of *EGFP* and *Xba*IR were normalized to human *GAPDH* or mouse *Gapdh* expression.

### Measurements of cellular bioenergetics

An Oroboros Oxygraphy-2 k (Oroboros Instruments, Innsbruck, Austria) was used to measure cellular bioenergetic changes in cells, as described previously^36^. In brief, the cells were harvested in 1 × 10^6^ cells/2 ml culture media. The cell suspension was transferred to a well, and Oroboros oxygraphy-2 k was used. After baseline measurements, oligomycin (2 μg/ml) as a complex V inhibitor, carbonyl cyanide-p-trifluoromethoxyphenylhydrazone (FCCP, 1 μM) as an uncoupler, a cocktail of rotenone (0.5 μM) as a complex I inhibitor and antimycin A (2.5 μM) as a complex III inhibitor were sequentially added to each well. Data are expressed as the oxygen consumption rates (O_2_ flow per cell; pmol/sec/cell). Routine, basal respiration, ETS, free routine activity, ROX, proton leakage and routine coupling efficiency were calculated as described previously. Seahorse XFe96 extracellular flux analyzer (Agilent Technologies Inc., Santa Clara, CA USA) was used to measure complex I changes in MirCs of 7S fibroblasts^23^. In brief, cells were seeded on XFe96‐well microplates. After 8‐hour incubation, the cells were washed and returned to the culture incubator until the assay was performed. The cells were washed twice and resuspended in 200 μl of unbuffered DMEM supplemented with 10 mM glucose, 2 mM glutamine and 1 mM sodium pyruvate (Agilent Technologies Inc.) and 10 mM disodium succinate hexahydrate (pH 7.4) (Sigma-Aldrich). The cells were equilibrated in a non‐CO_2_ incubator for 60 min prior to the assay. After three baseline measurements, oligomycin (1.5 μM), carbonyl cyanide p‐trifluoromethoxyphenylhydrazone (FCCP, 1 μM), rotenone (0.5 μM) and antimycin A (0.5 μM) were sequentially added to each well. Data are expressed as the oxygen consumption rates (OCR; pmol/min).

### Time-lapse fluorescence microscopy and high-resolution microscopy

To observe DsRed2-mitochondrial uptake into cells and cell growth, time-lapse fluorescence microscopy was performed using the JuLI stage (NanoEnTek Inc.). Red fluorescent images and phase images were taken every 30 min for 144 h from the beginning of mitochondrial coincubation. Fluorescence intensity was quantitatively analyzed to examine the fate of the fluorescent probes, and cell area (confluency) was automatically measured for the surrogate value of the cell number by using JuLI STAT software (Ver. 2.0.0.0, https://www.julistage.com, NanoEnTek Inc.). High-resolution microscopy of mitochondria was performed using N-SIM S (Nikon Corporation, Tokyo, Japan).

### iPS cell generation

Five days after mitochondrial transfer, the cells were seeded on 6-well plates at 1 × 10^5^ cells per well. The next day, four Yamanaka factors (OCT3/4, SOX2, KLF4, and c-MYC) were introduced into cells by Sendai reprogramming vectors. At day 6, the cells were harvested by trypsinization and plated onto feeder-MEF (ReproCELL, Kanagawa, Japan) at 6 × 10^5^ cells per 60 mm dish. After 24 h, the medium (DMEM containing 10% FBS) was replaced with iPS cell culture medium for primate embryonic stem (ES) cell medium (ReproCELL) supplemented with 20 ng/ml basic fibroblast growth factor (bFGF: Fujifilm Wako Pure Chemical). Approximately two weeks later, some granulated colonies appeared. At day 23, ES cell-like colonies were picked with a 200 μl tip and transferred to a 6-well plate containing adhered feeder-MEF.

These colonies were stained using the BCIP/NBT Substrate System (Agilent Technologies, Santa Clara, CA, USA) according to the manufacturer’s recommendations. Alkaline phosphatase (AP)-positive colonies were counted using ImageJ (National Institutes of Health, Bethesda, MD, USA).

Well-grown colony lines, such as ES cells, were maintained by changing the iPS culture medium daily and passaging the cells every 5–6 days. The cells were harvested with CTK solution [2.5% trypsin, 5 ml of 1 mg/ml collagenase-IV (Thermo Fisher Scientific incorporated), 500 μl of filtered (0.2 μm) 0.1 M CaCl_2_, and 10 ml of Knockout Serum Replacement (KSR: Thermo Fisher Scientific Incorporated)]. The harvested cells were transferred to 15 ml conical tubes and centrifuged at 160 × g for 5 min. The supernatant was carefully discarded without disturbing the cell pellet. Then, the cells were resuspended in fresh iPS culture medium and mixed twice to three times with soft pipetting. Slightly crushed colonies were plated onto a new feeder-MEF-coated 60 mm dish. The feeder-MEFs were seeded at 3 × 10^5^ cells per gelatin-coated 60 mm dish on the day before passage.

### Immunocytochemistry

The cells were fixed with 4% paraformaldehyde at 4 °C for 5 min and permeabilized with 0.1% Triton X-100 at room temperature for 20 min in the presence of a protein-blocking solution consisting of PBS supplemented with 5% normal goat serum (Agilent Technologies, Inc., Santa Clara, CA, USA). The cells were incubated overnight with primary antibodies in PBS at 4 °C. They were washed extensively in PBS and incubated at room temperature for 30 min with secondary antibody. The nuclei were counterstained with 4’,6-diamidino-2-phenylindole (DAPI; diluted 1:500; FUJIFILM Wako Pure Chemical) in PBS at room temperature for 30 min. To prevent fading during microscopy, the cells were mounted in DakoCytomation Fluorescent Mounting Medium (Agilent Technologies, Inc.). Immunofluorescence images were visualized and recorded using a Biorevo BZ-9000 fluorescence microscope (Keyence Corporation, Osaka, Japan).

### Evaluation of plasmid transfection efficiency

he transfection efficiency of the plasmid was quantified by calculating the expression rate of EGFP. The expression of EGFP was measured before and after treatment with puromycin treatment for 24 h. After 30 min of incubation in medium supplemented with Hoechst 33342 (Dojindo Molecular Technologies, Inc., Kumamoto, Japan), fluorescent images were captured at 3 random points using an IX71 fluorescence microscope (Olympus, Tokyo, Japan). Transfection efficiency was determined by the ratio of EGFP-positive cells to Hoechst 33342-positive cells.

### Immunoassay and flow cytometric analysis

Six days after plasmid transfection, we performed a protein immunoassay using antibodies against AMPK, pAMPK, S6, and pS6 on a Wes system (ProteinSimple, Inc., San Jose, CA, USA). We compared the activation levels of AMPK and S6 in ρ(-) NHDFs with other conditions: rapamycin, phosphatidic acid and starvation. Briefly, cells were treated with 50 nM rapamycin (Merck Millipore, Billerica, MA, USA) or 20 µM phosphatidic acid (Avanti Polar Lipids, Alabaster, Alabama, USA) for 24 h prior to sample collection. Starvation was performed by incubation with amino acid-free DMEM (Fujifilm Wako Pure Chemical) for one hour before sample collection.

For the immunoassay, treated cells were washed with PBS and collected in RIPA buffer (Fujifilm Wako Pure Chemical) containing protein inhibitor (Sigma-Aldrich). The cells were incubated on ice for 30 min and then homogenized. The extracts were spun down at 10,000 × g for 10 min at 4 °C, and the supernatants were analyzed using a Bio-Rad protein assay kit (Bio-Rad Laboratories, incorporated). The protein concentration of all samples was 0.2 mg/ml, and the primary antibody was diluted 50 times. The primary antibodies used in this experiment are listed in Supplementary Table S1.

For flow cytometric analysis, cells were washed with PBS, harvested with 0.25% trypsin–EDTA and subjected to FCM analysis. The DsRed2-positive cell population was evaluated using 488 and 561 nm lasers. Fluorescence data were collected using SH800S (Sony). The flow cytometry files were analyzed using FlowJo software (Ver. 10.6.1, https://www.flowjo.com/solutions/flowjo, Becton, Dickinson and Company).

### Evaluation of tolerance to H_2_O_2_ stimulation and starvation

We compared tolerance to H_2_O_2_ stimulation and starvation between each cell using the Annexin V-FITC Apoptosis Detection Kit (Nacalai Tesque, Kyoto, Japan). Cells were seeded in 6-well plates at 1 × 10^5^ cells per well. The next day, 600 µM H_2_O_2_ (Fujifilm Wako Pure Chemical) or amino acid-free DMEM (Fujifilm Wako Pure Chemical) was added to the cells. After 3 h H_2_O_2_ treatment or 48 h starvation, the cells were washed with PBS and collected in centrifuge tubes. Annexin V-FITC and PI solution were added to the cells and allowed to react for 30 min at room temperature protected from light. Then, the cells were rapidly analyzed by FCM analysis using SH800S (Sony) and FlowJo software (Becton, Dickinson and Company).

### Short tandem repeats (STR) profiling

A GenePrint 10 System PCR Amplification kit (Promega Corporation) was used to determine the genetic signature of both samples based on the multiplex analysis of 9 loci and the Amelogenin sex-determining marker. PCR products were run in the Applied Biosystems 3730xl DNA Analyzer (Thermo Fisher Scientific Incorporated) and analyzed using GeneMapper ID Software (Ver. 4.0, https://www.thermofisher.com/order/catalog/product/4475073#/4475073, Thermo Fisher Scientific Incorporated) following the manufacturer's recommendations. TH01, D21S11, D5S818, D13S317, D7S820, D16S539, CSF1PO, AMEL, vWA, and TPOX are regions to be used in this experiment. The quantitative results were radically plotted and lineally connected each other to easily discriminate genotypes.

### Single-cell droplet digital PCR (sc-ddPCR)

The sc-ddPCR protocol commenced with the encapsulation of a single cell into one oil droplet and then proceeded to the PCR step with a set of primers and fluorescent probes, which were the same as those used in the TaqMan SNP genotyping assay mentioned above, using TaqMan Polymerase with a 5’ to 3’ exonuclease, which releases the fluorophore from the probe, followed by the detection of the fluorescent signal in the droplets. The PCR mixture consisted of 4 μl of resuspended cells at a concentration of 2.5 × 10^5^/ml or 1.25 × 10^5^/ml, 10 μl of 2 × ddPCR Supermix (Bio-Rad), wild-type and mutant allele-specific TaqMan probes at a concentration of 0.25 μM, primer mixtures at a concentration of 0.9 μM for the target gene, and nuclease-free water for a final volume of 20 μl. Droplets were generated using the Bio-Rad QX200 system (Bio-Rad) following the manufacturer’s instructions. The reactions were transferred to a 96-well plate (Eppendorf Corp., Hamburg, Germany) for PCR using a thermal cycler (Bio-Rad) under the following conditions: amplification was carried out at a regular ramp rate of 2.0 °C/s at 95 °C for 10 min followed by 40 cycles of 30 s at 95 °C plus 2 min at 56 °C. The final enzyme deactivation step occurred at 98 °C for 10 min. The 96-well plate was transferred to a QX200 Droplet Reader (Bio-Rad), and the number of fluorescent droplets was analyzed. Each droplet was analyzed individually using a two-color detection system (set to detect FAM and VIC). The fluorescent droplets were counted to provide an absolute quantification of target mtDNA in digital form using QuantaSoft software (Ver. 1.7.4.917, https://www.bio-rad.com/en-us/sku/1864011-quantasoft-software-regulatory-edition?ID=1864011, Bio-Rad).

### Statistical analysis

All calculations were performed and plots were created using Prism 8 (GraphPad Software Inc., San Diego, CA, USA). The results are expressed as the mean ± S.E. The statistical significance of differences between the groups was evaluated using Student’s t-test, and P-values < 0.05 were considered significant.

## Results

### Mitochondrial DNA replacement in normal human dermal fibroblasts

**Figure 1:**
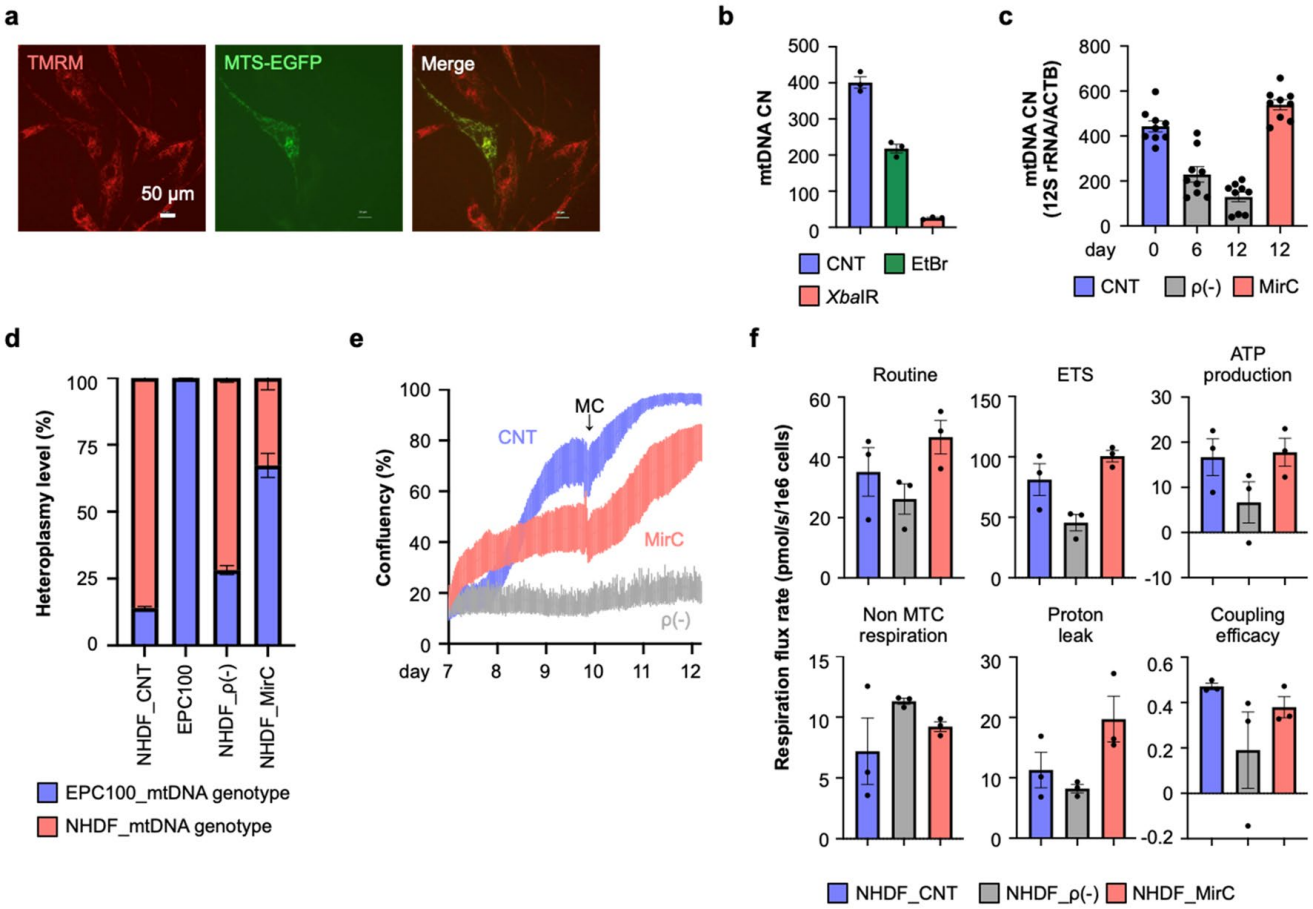
MirCs were generated from normal human dermal fibroblasts (NHDFs). (**a**) Left; TMRM staining specific for mitochondria in NHDFs, Middle; NHDFs, which were transfected with pCAGGS-MTS-EGFP-PuroR and selected with puromycin, reticularly expressed EGFP, Right; EGFP completely merged the red fluorescence of TMRM, verifying that MTS correctly transported downstream genes of interest to mitochondria. White bar indicates 50 µm. (**b**) By qPCR on day 5 following the treatment for mtDNA reduction, the mtDNA copy number (CN) of endonuclease-treated cells was decreased to less than 10%, clearly contrasted with the effect of EtBr treatment. (n = 3, respectively). (**c**) MtDNA CN in MirC increased to baseline levels on day 12 with coincubation of isolated mitochondria. (n = 9, respectively). (**d**) Heteroplasmy in MirCs of NHDFs with exogenous mitochondria showed the domination of exogeneous origin of mtDNA with a minor portion of the endogenous genotype. (n = 3, respectively). (**e**) Although the proliferation of MirCs was lower than that of the parental cells from day 7 to day 10 following mitochondrial DNA replacement, that of MirCs was equivalent to that of the parental cells after day 10, when medium change (MC) was performed. ρ(-) cells proliferated quite slowly throughout all periods. The cell numbers were quantified and plotted by JuLI STAT software based on the area of cells. MC: medium change. (**f**) Key parameters of respirometry were quantified. (n = 3, respectively) MTS; mitochondrial transfer signal. NHDF: normal human fibroblasts. EtBr: ethidium bromide. CNT: no treatment NHDF. ρ(-): rho minus indicates cells with a low mtDNA number. CN: copy number. ETS: maximum respiration of electron transport system.

To significantly reduce the mitochondrial genome, a plasmid carrying *Xba*IR, an endonuclease with a mitochondrial transfer signal, was designed (Supplementary Fig. S1a). A standard sequence of human mtDNA, referred to as the Cambridge reference sequence (CRS), has five *Xba*IR endonuclease recognition sites (Supplementary Fig. S1b). The capability of *Xba*IR to digest human mtDNA was verified in vitro (Supplementary Fig. S1c). Mitochondria have a poor capability to repair DNA breaks, and nucleic acid fragments are degraded in the mitochondrial matrix^31^. Among the mitochondrial transfer signals that have been reported, we selected the COX8A signal sequence in this study (Supplementary Fig. S1d), and the ability to transfer its fusion protein with EGFP to mitochondria was verified in normal human dermal fibroblasts (NHDFs). The recombinant plasmids were transfected into NHDFs with an electroporator and stained with tetramethylrhodamine methyl ester (TMRM), which is a fluorescent dye that accumulates in mitochondria. EGFP completely merged the fluorescence of TMRM (Fig. 1a). The transferred *Xba*IR effectively reduced the content of mitochondria in immortalized human EPC100 cells, which genetically labeled mitochondria with DsRed2, 5 days after gene transfer (Supplementary Fig. S1e–g). The endonuclease was better than ethidium bromide (EtBr),which has been used for ρ0 generations in mitochondrial reduction. The mtDNA CN, which was estimated by the quantification of 12S ribosomal RNA unique for mtDNA, declined to less than 10% of the original content on day 5 following gene transfer, and this cellular state was termed ρ(-) cells (Fig. 1b). Puromycin exposure for 24 h efficiently enriched the gene-transferred cells (Supplementary Fig. S1h, i). On day 12 following the gene transfer of *Xba*IR, the mitochondrial membrane potential, a surrogate marker of mitochondrial genome integrity, showed a clear decrease in NHDFs (Supplementary Fig. S1j). The mRNA expression analysis of *Xba*IR to be transferred showed time-dependent expression with a peak on day 2 and a rapid decrease to undetectable levels on day 14 (Supplementary Fig. S1k).

The protocol to generate MirCs, which constitutes a 1-day drug selection on day 2 and cocultivation with isolated exogenous mitochondria on day 6, was designed (Supplementary Fig. S2a). On day 12 following coincubation with isolated mitochondria, the content of mitochondria recovered to the original level (Fig. 1c). MirCs of NHDFs with EPC100 mitochondria were examined by sequencing short mitochondrial genome fragments to detect the origin of mtDNA (Supplementary Fig. S2b). Both nucleotides originating from EPC100 cells and NHDFs were significantly present in MirCs on day 12, although PCR-directed sequencing could not be quantitatively analyzed (Supplementary Fig. S2c). A TaqMan single nucleotide polymorphism (SNP) genotyping assay was applied to quantify heteroplasmic mtDNAs based upon the difference at position 16,362 (NHDF: A, EPC100: G) in the D-loop (Supplementary Fig. S2d). The rates of the genotype of G in m16362 of NHDFs and EPC100 cells were approximately 10% and almost 100%, respectively. MirCs contained an approximately 70% exogenous genotype in mtDNAs (Fig. 1d), suggesting that this protocol could revert the heteroplasmy of cells in mitochondrial diseases.

### Metabolic recovery of MirC from ρ(-) cells

The phenotypic recovery of MirCs of NHDFs was demonstrated with respect to cell proliferation, while ρ(-) cells of NHDFs showed a poor proliferative capability (Fig. 1e). Whether the transferred mtDNA could generate energy was investigated with a coupling control protocol by using high-resolution respirometry. Representative oxygen consumption rate curves of native cells, ρ(-) cells, and MirCs on day 12 of the protocol to generate MirCs are shown, and the respiratory flow and control ratio were calculated (Supplementary Fig. S2e; Fig. 1f). Routine respiration, electron transfer system (ETS) maximum capacity, ATP production, and coupling efficiency showed the same trends: the indexes were decreased in ρ(-) cells compared with parental cells and increased in MirCs, approaching the levels in parental cells. Proton leakage was increased following mtDNA replacement compared with the native and ρ(-) cells, which might reflect mitochondrial membrane damage by mtDNA reductions. These results confirmed that this protocol can enable the replacement of mtDNA in somatic cells with clinically applicable materials and procedures.

### Mitochondrial DNA replacement in fibroblasts in a mitochondrial disease patient Leigh syndrome

**Figure 2:**
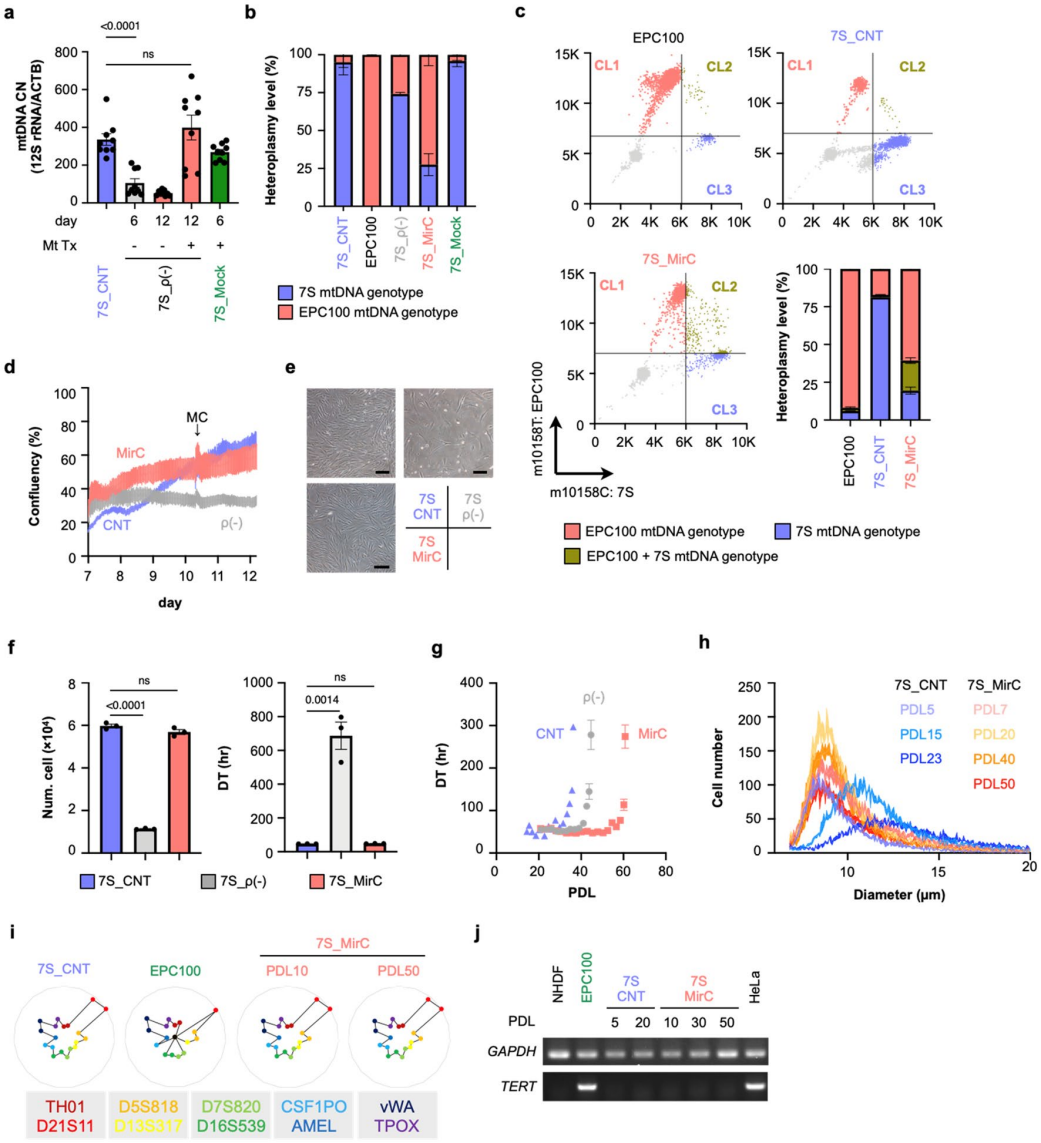
MirCs were generated from mitochondrial disease patient-derived (7S) fibroblasts. (**a**) mtDNA CN during the procedure of MirC generation. Fibroblasts that received gene transfer, designated as 7S_ρ(-) were cultivated with or without isolated mitochondria. Mock transfectants that received a plasmid without the endonuclease, designated as 7S_Mock, were subjected to the same protocol. (n = 9, respectively). (**b**) TaqMan qPCR SNP genotyping assay demonstrated the dominance of exogenous mtDNA. MirCs derived from 7S fibroblasts were designated as 7S_MirC. (n = 3, respectively). (**c**) Heteroplasmic sc-ddPCR discriminated three different populations: healthy homoplasmic cells (Cluster 1: CL1, red), heteroplasmic cells (CL2, brown), and mutated homoplasmic cells (CL3, blue) for mtDNA. Representative analyses are shown in the quadrant plotting, and the averages are depicted as a bar graph. Donor mitochondria for MirCs were isolated from EPC100 cells. (n = 3, respectively). (**d**) Cell growth of MirCs compared with the original cells and ρ(-) cells by using time-lapse imaging recorder from day 7 to day 12 in the protocol. The confluency was automatically calculated by JuLI STAT software. (**e**) Microscopic photographs of cell cultures following mitochondrial replacement 5 days after replating at a concentration of 1 × 10^5^ cells on day 12 in the protocol. (**f**) The yield of cells and the doubling time of MirCs were similar to those of 7S fibroblasts. The black bar indicates 200 µm. (n = 3, respectively). (**g**) Long-term culture showed the lifespan extension of MirCs. (n = 3, respectively). (**h**) The cell size of MirCs was maintained during culture, whereas that of the original cells was significantly enlarged from early PDL with time. (n = 3, respectively). (**i**) Short tandem repeats (STRs) demonstrated no contamination of the original MirCs by EPC100 cells that provided the donor mitochondria for MirCs. (**j**) TERT expression in MirCs to deny carcinogenic transformations. The full-length gel of cropped gels is shown in Supplementary Fig. S4. mtDNA, mitochondrial DNA. CNT, no treatment control cell. ρ(-), rho minus, indicates cells with a low mtDNA number. CN, copy number. MC, medium change. DT, doubling time. PDL, population doubling level.

We next attempted to correct cells derived from patients with mitochondrial disease with mtDNA mutations by using the somatic mtDNA replacement protocol. We used primary fibroblasts derived from a patient diagnosed with Leigh syndrome with a T10158C mutation, which is in the NADH-ubiquinone oxidoreductase chain 3 (ND3) locus of complex I, when she was 3 months old, named 7S fibroblasts (Supplementary Fig. S3a, b). The same mtDNA replacement protocol performed with NHDFs was applied to 7S fibroblasts. The sequencing of mtDNA at the 10158th nucleotide showed T in the donor mitochondria derived from EPC100 as CRS (Supplementary Fig. S3a, b). The mtDNA CN assay showed that the kinetics following the mtDNA replacement protocol in 7S fibroblasts were almost the same as the kinetics observed in NHDFs (Fig. 2a). Differences were observed in the reduction rate, which was decreased significantly more in 7S fibroblasts than in NHDFs both day 6 and day 12 in the mtDNA replacement protocol. The mtDNA content recovered to the baseline level in the original 7S fibroblasts on day 12. Whether the mitochondria in 7S fibroblasts contain exogeneous mtDNA was examined by sequencing the mitochondrial genome fragment including the 10158th nucleotide. Large waves of T and small waves of C were observed in MirCs that received mitochondrial replacement (Supplementary Fig. S3c). We performed a TaqMan SNP genotyping assay to estimate heteroplasmy through this intervention (Supplementary Fig. S3d). The original heteroplasmy in 7S fibroblasts was more than 90%, whereas the MirCs derived from 7S fibroblasts exhibited approximately 25% heteroplasmy on day 12 in the protocol (Fig. 2b). To examine the necessity of endonuclease treatment, mock transfectants of 7S fibroblasts were cocultured with isolated mitochondria of EPC100 cells, resulting in no change in heteroplasmy (Fig. 2b, Supplementary Fig. S3e). We investigated heteroplasmy in single cells by using single-cell droplet digital PCR (sc-ddPCR) on day 12 in the protocol, which enabled multiplex detection for the presence or absence of a particular genotype in a cell (Fig. 2c)^15^. This assay, the results of which were plotted in quadrant analysis format by using FlowJo software for FACS analysis, revealed that homoplasmic cells with either healthy or mutated mtDNA were present in 7S fibroblasts. In 7S fibroblasts, homoplasmic cells with mutated mtDNA were more than 75%, and healthy homoplasmic cells were approximately 20%. MirCs of 7S fibroblasts to be generated with healthy mtDNA by our protocol exhibited that homoplasmic cells with healthy mtDNA became dominant over 60%.

MirCs derived from 7S fibroblasts regained the proliferation capability compared with ρ(-) cells, although the proliferation rate of the MirCs was still less than that of the parental 7S fibroblasts until day 12 (Fig. 2d). After replating cells on day 12, the morphology and the doubling time in the MirCs of 7S fibroblasts became the same as that in the parental cells (Fig. 2e,f). For long-term culture, MirCs of 7S fibroblasts demonstrated dramatic lifespan extension up to the 63rd population doubling level (PDL), given that growth arrest is defined as the time when the doubling time is more than 120 h (Fig. 2g). The cells received mitochondrial replacement at the 8th PDL so that the reconstituted cells with healthy mtDNA might have a lifespan up to the 55th PDL, which is the Hayflick limit in cell culture. Due to mitochondrial dysfunction in 7S fibroblasts, they exhibited a larger cell size with time (Fig. 2h). The diameter of 7S fibroblasts was approximately 1.5 times larger than that of NHDFs by the 15th PDL and increased by an additional 2 times by the 23rd PDL, so the volume was 3 to 8 times larger. In line with the proliferation recovery after mitochondrial replacement in 7S fibroblasts, the cellular sizes of MirCs of 7S fibroblasts were maintained over time (Fig. 2h). The contamination of the donor cells into MirCs was denied by using a short tandem repeat (STR) assay (Fig. 2i, Supplementary Table S2). The tumorigenesis of MirCs was also denied by measuring the expression of TERT as a tumor marker (Fig. 2j, Supplementary Fig. S4).

### Phenotypic recovery in MirCs derived from fibroblasts of a mitochondrial disease patient

**Figure 3:**
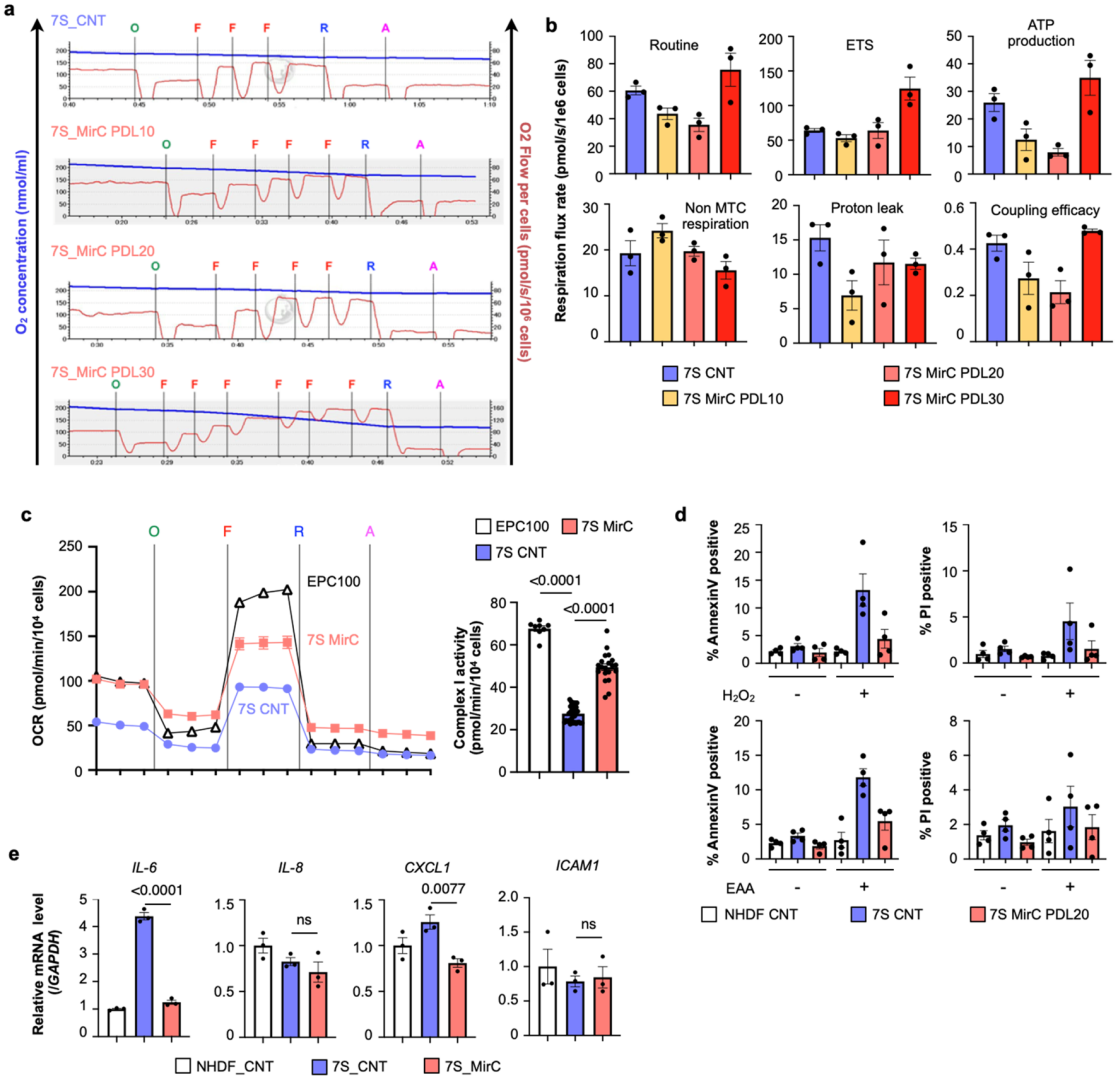
Phenotypic characterization of MirCs. (**a**) Respiratory functions of MirCs at PDLs 10, 20, and 30 following mtDNA replacement by using a coupling control protocol (CCP) in Oroboros O2k. O; 4 mg/ml oligomycin. F; 1 mM carbonyl cyanide 4-(trifluoromethoxy)phenylhydrazone (FCCP). R; 1 mM rotenone. A; 5 mM antimycin. (n = 3, respectively). (**b**) Respiratory flow rates demonstrated that it took approximately 30 PDLs to improve respiratory function. (**c**) Respiratory chain complex I activities estimated by respirometry. The activities in 7S fibroblasts (n = 34) were depressed, whereas the activities of MirCs were regained (n = 19). As a positive control, EPC100 cells that were mitochondrial donor were examined. (**d**) Mitochondrial disease patient-derived fibroblasts were more sensitive to H_2_O_2_ and essential amino acid (EAA)-free medium than their MirCs. MirCs of 7S fibroblasts suppressed late apoptosis, which were measured as PI positive rates, at levels similar to those of NHDFs. Early apoptosis, which were measured as annexin V positive rates, in MirCs was also significantly ameliorated in 7S fibroblasts (n = 4). (**e**) Representative factors of the senescence-associated secretory phenotype of 7S fibroblasts and MirCs of 7S fibroblasts. CNT, no treatment control cell. PDL, population doubling level. ETS, electron transport system. Non-MTC respiration, nonmitochondrial respiration. (n = 3, respectively).

MirCs derived from 7S fibroblasts were investigated by using the coupling control protocol (CCP) of Oroboros O2k with respect to respiratory function over time after the transfer (Fig. 3a). Routine respiration and free routine activity (ATP production) decreased by the 20th PDL after replacement, and the maximum capacity of the electron transport system (ETS) maintained the original levels of 7S fibroblasts. On the 30th PDL after replacement, all three indexes with respiratory function strikingly ascended and surpassed the original (Fig. 3b). These results indicate that it takes a given time period to reconstitute the electron transfer system with healthy and nonmutated complex I following mitochondrial DNA replacement. In the early phase after replacement, a reduction in mutated mtDNA might have exposed a more critical energy shortage than that in experiments using NHDFs; consequently, the delay in recovery might have emerged. Nonmitochondrial respiration decreased over time after replacement. The reason why the change occurred earlier than that of ATP production might be related to the quick recovery in cell size following mitochondrial DNA replacement (Fig. 2g). Leak respiration, which reflects intrinsic uncoupling, such as proton leakage, proton slip, and electron slip, decreased during a shortage of ATP production in mitochondria and maintained low values at the time when the reconstitution was likely complete. The kinetics of the coupling rate were the same as the kinetics of ATP production, consistent with the strong relationship between the coupling rate and ATP production. The functional reversal on the 30th PDL after replacement, which was comparable with that of NHDFs, indicates the clue of the strategy for clinical applications. We evaluated the activities of respiratory chain complex I, which is expressed as the difference between the oxygen flows of basal respiration and those after administrating rotenone^37^. Native 7S fibroblasts with a mutation of ND4 in complex I showed a disturbance in oxygen consumption in complex I, whereas MirCs derived from 7S fibroblasts regained the respiration in complex I, indicating that complex I in MirCs was reconstituted by the wild-type ND3 protein encoded in exogenous mtDNA (Fig. 3c).

The stress responses of 7S fibroblasts were examined with an oxidative stress model exposed to H_2_O_2_ and a starvation model cultivated in essential amino acid-free medium to evaluate apoptosis. By using Annexin V as an early apoptosis marker for phosphatidyl serine and propidium iodide as a late apoptosis or necrosis marker, the levels of both stresses, in which NHDFs were tolerated and 7S fibroblasts were significantly damaged, resulting in apoptosis, were examined to examine the stress response of MirCs (Fig. 3d). MirCs derived from 7S fibroblasts were protected from both stresses, although the early apoptosis in MirCs was higher than that in NHDFs but lower than that in 7S fibroblasts. MirCs derived from 7S fibroblasts showed restored IL-6 and CXCL1 expression similar to that of NHDFs (Fig. 3e). These results might suggest that sensitivity to cell death and progeria phenotype in 7S fibroblasts could also be reverted to normal through mtDNA replacement.

### Kissing exogeneous mitochondria with endogenous mitochondria to transfer donor mtDNA

**Figure 4:**
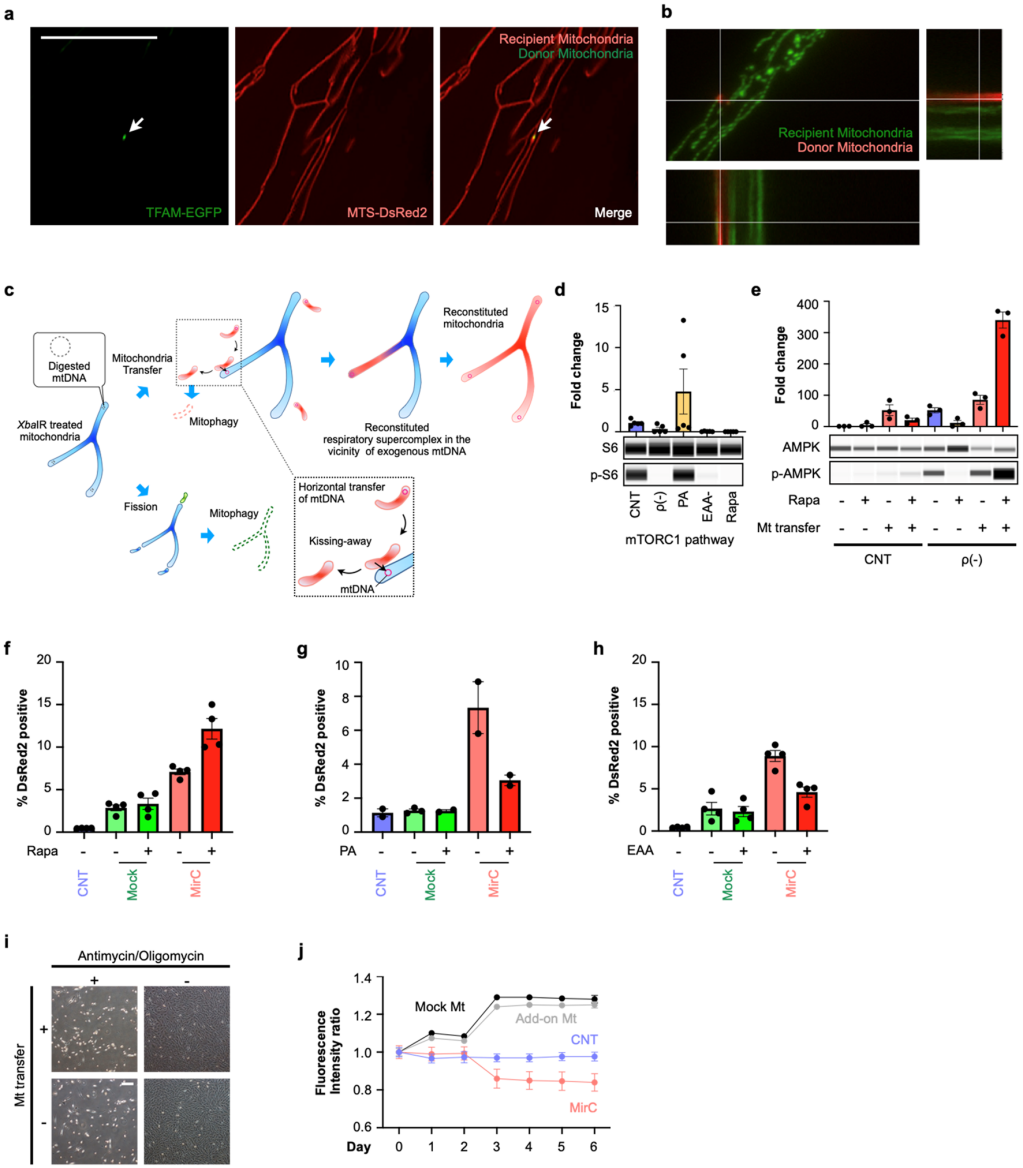
Mechanistic insights into the process of mtDNA replacement. (**a**) Superfine microscopy showed that exogenous mtDNA coupled with TFAM, which was genetically fused with EGFP, existed in endogenous mitochondria, which were genetically marked with DsRed. White bar indicates 50 µm. (**b**) Fibroblasts with EGFP marked mitochondria were subjected to MirC generation protocol with DsRed-marked mitochondria. Following the coculture, two fluorescence signals were always far apart and showed temporary contact for a short period. (**c**) A model of “Kissing away” in transferring mtDNA into endogenous mitochondria. (**d**) Activation of mTORC1 was measured by the ratio of phosphorylation of S6 to S6. ρ(-) cells derived from NHDFs significantly decreased the mTORC1 pathway, similar to that observed in essential amino acid-free medium and rapamycin treatment. (n = 5, respectively). (**e**) AMPK activation during the MirC generation protocol with rapamycin. (n = 3, respectively). (**f**) Rapamycin enhanced the engulfment of exogenous mitochondria in the MirC generation protocol. (n = 3, respectively). (**g**) PA suppressed macropinocytosis in the MirC generation protocol. (n = 3). (**h**) EAA free media suppressed the engulfment of exogenous mitochondria in MirC generation protocol. (n = 3). (**i**) ρ0 cells that completely lacked mtDNAs were generated by adding antimycin and oligomycin to Parkin-overexpressing cells and showed no engulfment of exogenous mitochondria. White bar indicates 200 µm. (**j**) The fate of exogenous mitochondria. Fluorescent area was measured as a surrogate for the fate of exogeneous mitochondrial membranous components. Mock transfectants or cells that were cocultured with isolated mitochondria without mtDNA reduction, designated as add-on, showed the gradual increased intensities, whereas cells subjected to MirC protocol showed diminusion. (n = 3) The full-length gels of cropped gels in d and e are in Supplementary Fig. S6 and S7, respectively. Mock: Mock transfectants that received a plasmid without XbaIR sequence.

The fate of the mitochondria transferred into cells was investigated separately on nucleoids including mtDNA or mitochondrial protein components (Supplementary Fig. S5a, b). Mitochondrial transcription factor A (TFAM), which binds to mtDNA and regulates mitochondrial biogenesis, was selected to trace exogeneous mitochondrial nucleoids. To investigate the fate of the donor mitochondrial nucleoids, genetically TFAM-marked EPC100 cells were created by infecting the recombinant retrovirus carrying the sequence encoding a fusion protein of TFAM and EGFP (Supplementary Fig. S5c). TFAM-marked mitochondria were used as donors, and DsRed2-marked NHDFs in the mitochondrial matrices were used as recipients. During the mtDNA replacement protocol, cells were observed by hyperfine microscopy. The donor nucleoids settled in the pre-existing mitochondrial matrices (Fig. 4a). In the combination of DsRed2-marked mitochondria derived from EPC100 as donors and ρ(-) cells derived from genetically mitochondria-targeted EGFP-marked NHDFs as hosts, the fate of mitochondrial protein components was examined using superfine microscopy (Fig. 4b). The contacts of the donor and the resident mitochondria were recognized, but no broad fusion was observed (Supplementary Movies 1-1 to 1-4). Exogenous mitochondria contacted the pre-existing mitochondria for a short period then disappeared soon after leaving away endogenous mitochondria, such as kissing away. Moreover, the fluorescent spots of donor mitochondria on day 2 were quite rare, suggesting that exogeneous mitochondrial protein components were degraded for a short time.

We hypothesized a model to transfer mtDNA in this protocol. In the model, exogeneous mitochondria contact with preexisting mitochondria that had lost most mtDNA, and then exogenous mtDNA are transferred to the preexisting mitochondrial matrices, whereas exogenous vacant mitochondria are degraded (Fig. 4c). Since our results did not cover all events of exogenous mtDNA to settle down in the host cells, this model that we consider the most conceivable remains to be further examined.

### Macropinocytosis of exogeneous mitochondria is regulated by the mTORC1 pathway

We investigated the mechanism that regulates the macropinocytosis of exogeneous mitochondria to create an ideal protocol to generate MirCs of any type of cell. Since ρ(-) cells are exhausted of ATP to support cellular activities, the intracellular energetic state of ρ(-) cells could be compatible with starvation with respect to ATP content, but ρ(-) cells in a cell culture medium sense nutrient replete. Essential amino acid (EAA)-free medium for AMPK stimulation, phosphatidic acid (PA) for mTORC1 activation, and rapamycin for mTORC1 suppression were chosen to examine the state of ρ(-) cells. The ratios of phosphorylated AMPK to AMPK and phosphorylated p70 S6 kinase to p70 S6 kinase, which is a downstream target of mTORC1, were examined by using capillary electrophoresis, namely, Wes (Protein Simple). The mTORC1 pathway was drastically suppressed in ρ(-) cells to the same level as that observed under starvation conditions or rapamycin treatment (Fig. 4d, Supplementary Fig. S6). ρ(-) cells activated the AMPK pathway, the coincubation of exogeneous mitochondria enforced the activation, and the addition of rapamycin further strengthened the activation (Fig. 4e, Supplementary Fig. S7). We examined the effects of rapamycin, PA, and free EAA on mitochondrial engulfment during mitochondrial cocultivation. At 48 h following the coincubation with isolated DsRed2-marked mitochondria, FACS analyses were performed to detect DsRed2. Rapamycin significantly augmented the engulfment of the mitochondria in ρ(-) cells to generate MirCs (Fig. 4f). On the other hand, PA clearly suppressed it (Fig. 4g). Depletion of EAA ameliorated the engulfment of ρ(-) cells, contrary to expectations (Fig. 4h). In mock transfectants, no treatment had a significant impact on the engulfment of exogenous mitochondria. These results suggest that mTORC1 could be a regulator of mitochondrial macropinocytosis in ρ(-) cells.

Whether the mtDNA replacement protocol can be refined through the complete elimination of endogenous mtDNA, named ρ0 cells^38^ was examined by using cells genetically modified to overexpress PARKIN, which is a key molecule for mitophagy (Fig. 4i). The transfectants could generate ρ0 cells by administrating oligomycin and antimycin, and the ρ0 cells could not engulf exogeneous mitochondria with poor mobility (Supplementary Movies 2-1 to 2-4). ρ(-) cells derived from NHDFs (Supplementary Movies 3-1) and 7S fibroblasts (Supplementary Movie 4) markedly engulfed the isolated mitochondria, as observed by time-lapse microscopy. However, both naïve NHDFs (Supplementary Movie 3-2) and mock-transfected NHDFs (Supplementary Movie 3-3) seemed to gather the isolated mitochondria, forming very large aggregates on the cell surfaces. The decline in engulfed exogeneous mitochondria started as soon as 72 h following coincubation in the mtDNA replacement protocol (Supplementary Movie 3-1), whereas the fluorescence of exogeneous mitochondria in either the mock transfectants or mitochondria add-on protocol increased rather than decreased (Supplementary Movie 3-2, 3-3, and Fig. 4j). A partial reduction in mtDNA could be essential for this protocol because a complete reduction inhibits macropinocytosis of exogeneous mitochondria, and add-on mitochondrial coincubation does not efficiently execute macropinocytosis.

### No reversion of heteroplasmy in induced pluripotent stem cells derived from MirCs

**Figure 5:**
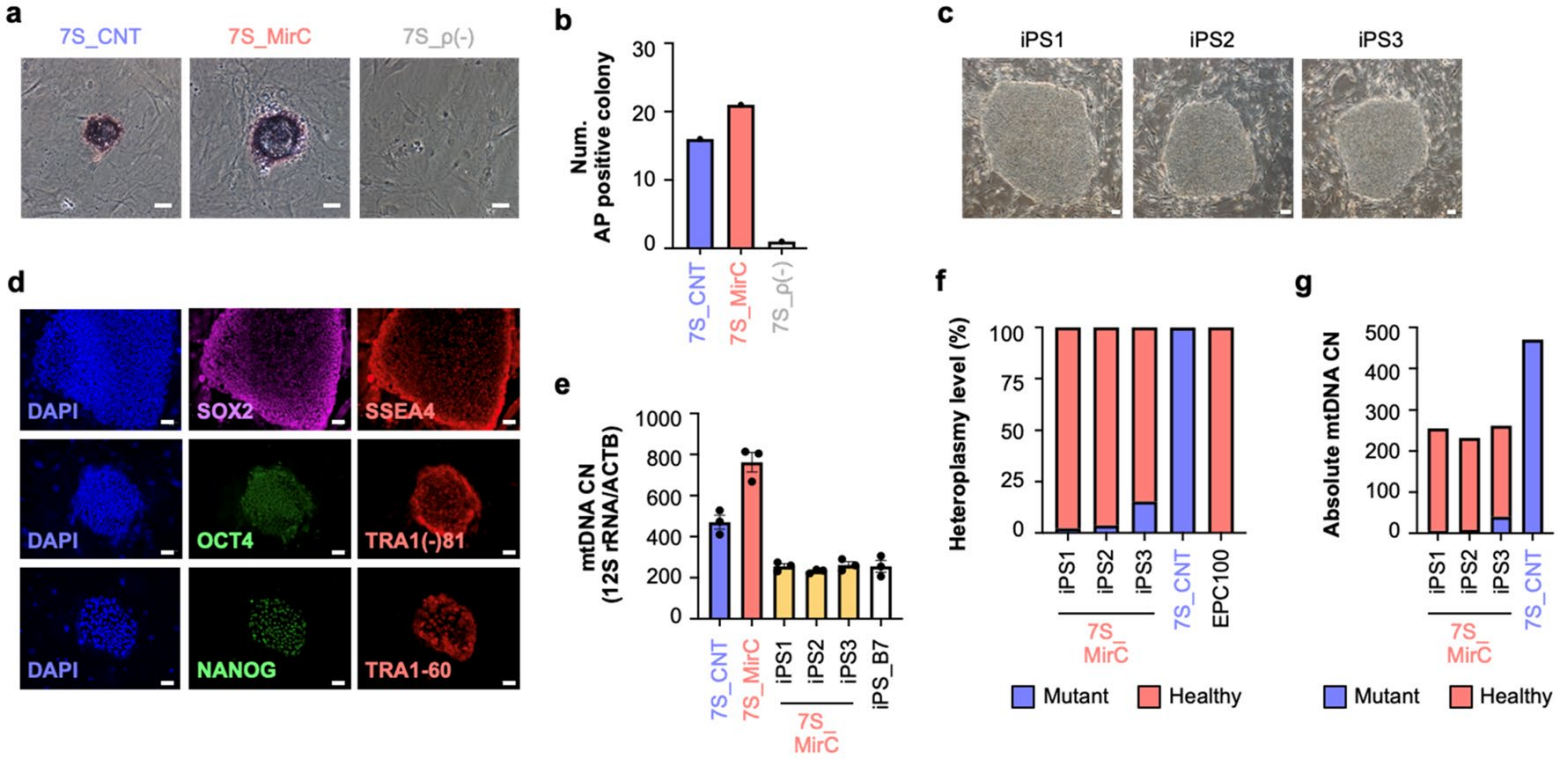
Characterization of induced pluripotent stem cells derived from the MirCs of mitochondrial disease patient fibroblasts. (**a**) Alkaline phosphatase (AP) assay at day 10 following the gene transfer to generate iPS cells showed vulnerable small colonies in 7S fibroblasts (7S_CNT), whereas MirCs (7S_MirC) with healthy mtDNA of 7S fibroblasts demonstrated firm colonies. The ρ(-) cells of 7S fibroblasts (7S_ρ(-)) did not generate any colonies. White bar indicates 50 µm. (n = 1). (**b**) The numbers of AP-positive colonies were not very different from those of the original cells and MirCs. (**c**) Representative photographs of iPS colonies in the long term. White bar indicates 100 µm. (**d**) Expression of stem cell markers, such as SOX2, OCT3/4, NANOG, SSEA4, TRA1-81, and TRA1-60. White bar indicates 50 µm. (**e**) MtDNA CNs in iPS cells derived from MirCs of 7S fibroblasts. (n = 3, respectively). (**f**) Heteroplasmy in three lines of iPS cells maintained the original rate of MirCs (n = 1). (**g**) The absolute CNs of healthy and mutated mtDNAs were calculated. CN: copy number.

Although there are many reports on the successful generation of induced pluripotent stem (iPS) cells from cells derived from patients with mitochondrial diseases^39^, we could not generate iPS cells from 7S fibroblasts by using standard methods of Sendai virus^40^ carrying OCT3/4, SOX2, KLF4, and c-MYC, although early colonies positive for alkaline phosphatase assay were recognized in 7S fibroblasts and MirCs of 7S fibroblasts with similar numbers of colonies (Fig. 5a,b). We acquired several lines of iPS cells, which were validated by staining with OCT4, SOX2, NANOG, SSEA4, TRA1-81, and TRA1-60, only from MirC-derived 7S fibroblasts (Fig. 5c,d). These iPS cells possess approximately half the mtDNA content of the parental cells without any variability (Fig. 5e), consistent with reports that embryonic stem cells have a lower mtDNA CN than parental differentiated cells^41^. The MirC-derived iPS cells exhibited less than 10% heteroplasmy of mutated mtDNA, which is similar to the heteroplasmy in the parental MirCs of 7S fibroblasts on day 160 following gene transfer (Fig. 5f,g). The mtDNA replacement protocol in somatic cells did not show reversion, which is a concern in mitochondrial replacement therapy (MRT) in oocytes, in long-term cultivation and maintained the original heteroplasmy level.

## Discussion

We established a novel technology to replace mtDNA in somatic cells with mtDNA reductions by mitochondria-targeted endonuclease. Exogenous mitochondria encounter the first barrier, the plasma membrane, for internalization and take advantage of macropinocytosis of host cells. As the second barrier, how exogenous mtDNA escapes macropinosomes remains elusive. The molecular mechanism of the import of mtDNA into mitochondrial matrices is also unclear. What is most practical issue is how the genotype to be introduced in our protocol is maintained without a reversion by the pre-existing genotype. This technology has a significant advantage in enabling the replacement of the mitochondrial genotype, although some issues remain to be unveiled.

Isolated mitochondria are mainly engulfed via macropinocytosis^23^, although other processes are involved in the internalization of exogeneous mitochondria. It was reported that macropinocytosis and mTORC1 share signaling pathways and seem to coordinately contribute to cellular growth^42^. We focused on two molecular pathways, mTORC1 and AMPK; the former acts as an essential sensor of amino acids, energy, oxygen, and growth factors and a key regulator of protein, lipid, and nucleotide synthesis^43^; the latter is a sensor of AMP levels whose activation results in autophagy, mitochondrial biogenesis, glycolysis, and lipolysis^44^. Both pathways are involved in the uptake of extracellular nutrients. Activated mTORC1 negatively regulates macropinocytosis through insulin response substrate (IRS)^45^. One report showed increased macropinocytosis in Ras-overexpressing cancerous cells upon the addition of rapamycin^46^, whereas rapamycin was reported to suppress macropinocytosis in bone marrow-derived primary cells under replete nutrient conditions^47^. This inconsistency might be attributed to signals originating receptors of tyrosine kinase. Upon coculture of ρ(-) cells of primary fibroblasts and isolated mitochondria, rapamycin was an effective activator of macropinocytosis, suggesting that a shortage of energy could reverse the regulation of macropinocytosis. In line with this assumption, pharmacological activation of AMPK in macrophages increased macropinocytosis^48^. Modifications of the mTORC1 and/or AMPK pathways in this method for MirC generation could be targets for the optimization of the protocol.

Solutions of isolated mitochondria contain some damaged mitochondrial membrane fragments and genomes called mitochondrial damage-associated molecular patterns, which might induce innate immunity in the cytosol^49^. The engulfed mitochondria in macropinosomes might circumvent the activation of innate immunity, as engulfed apoptotic bodies in macropinosomes do not induce innate immunity^50^. Either escape to the cytosol or direct contact with pre-existing mitochondria are required to function in the new destiny. Following the internalization of mitochondria by macropinocytosis, how exogenous mitochondria break macropinosomal membranes has not yet been revealed, although our results indicate that exogenous mitochondria transfer their genome to endogenous mitochondria. As entrapped pathogens in endosomes break through the membranes of endosomes by using membrane fusion, pore formation, receptor binding, proteolytic processing, or intraluminal acidification^51^, exogenous mitochondria might utilize one of them, or membranes of macropinosomes might fuse with the outer membrane of endogenous mitochondria.

There is a concern of damage to isolated mitochondria by high Ca^2+^ concentrations for their transfer^52^. The presence of Ca^2+^ at high concentrations in mitochondria could lead to the destruction of mitochondria if they stayed in the culture media for a long time. The coculture provides swift internalization through macropinocytosis, as the videos in supplementary materials show. In our protocol, exogenous mitochondria did not function by themselves inside the host cells because the fluorescence to be delivered into donor mitochondria rapidly disappeared following internalization into the host cells (Fig. 4c). Exogenous mtDNA, which is imported into mitochondrial matrices upon brief contact of exogenous mitochondria with endogenous mitochondria and is transcribed with the pre-existing replication machinery, shows that 12S rRNA increases on day 6 after coculture (Fig. 1c) and plays a key role in generating MirCs. Therefore, some damage to the mitochondrial shell could not be an obstacle for MirC generation. In addition, genetic transfer inside the cytosol evokes horizontal genetic transfer between prokaryotes^53^, which might survive even after losing capability as an independent organism in ancient times.

It remains to be solved how replication of the mammalian mitochondrial genome is regulated for homeostasis and how heteroplasmy is propagated. Mitochondrial gene replacement in oocytes can provide offspring^54^, and mitochondrial replacement in pluripotent stem cells with mitochondrial dysfunctions can normalize their metabolism^55^. Although MRT has already been applied in the clinic^34^, concerns that the mitochondrial genotype can be reverted to the original in embryonic stem cells derived from the embryo receiving the technology have been raised^56^. A mechanistic study on reversion revealed that genetic drift could be the cause^57^. A significant number of these ES cell lines showed the gradual domination of karyoplast-associated mtDNA with time in culture to reach homoplasmy^57–59^, although oocyte karyoplasts carried over less than 1% in maternal spindle transfer of rhesus macaques^60^. On the other hand, iPS cells derived from MirCs of 7S fibroblasts showed the constant and dominant healthy genotype over 160 days. *C. elegans* demonstrated that the mitochondrial unfolded protein response (UPRmt) functions to maintain a heteroplasmy and propagates mutated mtDNA following a disturbance of the heteroplasmy to recover the original heteroplasmy^61^. Prior to the study, UPRmt was only considered to promote the functional recovery of damaged mitochondria^62^; however, it is now understood as a double-edged sword, relying upon the quality of mtDNA^63^. The preservation of mutated mtDNA might be a selfish aspect to survive. Although mammalian cells possess the same molecular machinery is currently unknown, the entity of the compatible machinery in mammals might be highly probable^64^.

In addition to mitochondrial gene editing using ZFN^32^ and TALEN^33^, a sophisticated study was recently presented that designed a split new type of cytidine deaminase (DddA) combined with TALE protein to recognize a defined DNA sequence in tandem of mitochondrial targeting sequence (MTS) and separately uracil glycosylase inhibitor (UGI) to prevent the conversion of uracil to cytidine, called DdCBE^65^. One concern is that DdDBE reduced the mtDNA CN, as MirC generation takes a period to reconstitute a new mitochondrial complex and network (Fig. 3b). When this technology is applied in vivo, the cells that receive gene transfer might not be permissive due to a serious shortage of energy. In this DdCBE, uracil glycosylase inhibitor (UGI) functions to protect uracil to revert back to cytidine, leading to introduction of adenine in the counterstrands upon the next round of mtDNA replication, therefore theoretically changing the heteroplasmy to 50% at maximum. In addition, DdCBE can not target deletions or long-range replacement. MirC could provide advantages of superior heteroplasmy changes to dominate over the previous genotype and applicability to any mutation sequences, including deletions and long-range replacement, as well as no concern regarding off-target risks.

Mitochondrial augmentation therapy, which is provided by transplantation of hematopoietic stem cells that have been cocultivated with exogenous healthy mitochondria, has been conducting as a clinical trial for mitochondrial diseases in the United States several years ago^66^. Researchers that are involved in the trial showed mitochondrial transfer to HSCs by a simple coculture with isolated mitochondria, the extent of which seemed not to be so high to overwhelm endogenous mtDNA. Our procedure, which should be applicable to HSCs with mRNA transfection^67,68^ instead of a plasmid as CAR-T cells have been generated with mRNA transfection^69,70^, might provide better donor cells that are dominant for healthy mitochondrial genotypes. In addition to maternal inherited mitochondrial diseases, although mtDNA mutations do not causally contribute to physiological HSC aging^71^, accumulations of mtDNA mutations that are relevant to their dysfunction were recognized in fibroblasts and blood in age^72^. Immunometabolism governs immune cells upon differentiation fate and their functions, shaping the immune response^73^. Therefore, if MirCs can be generated from aged HSCs and immune cells, the MirCs could offer cell sources to investigate how mitochondria commit to the aging process in hematopoiesis and immunity.

This protocol offers drastic biological changes in human fibroblasts with mutated mtDNA through mitochondrial DNA replacement with healthy mtDNA. The replacement of mtDNA is stable in human fibroblasts without reversion, in contrast with mitochondrial replacement therapy in assisted reproductive technology. As a limitation, we demonstrated a protocol to achieve mitochondrial DNA replacement only in human fibroblasts, but not in other types of cells. Our next step is to demonstrate that MirCs derived from HSCs and immune cells can be generated. In addition, the accurate mechanism in mtDNA engraftment and circumvent of reversion to the original mitochondrial genotype is required for clinical application to treat maternal inherited mitochondrial diseases. At least, this technology can provide a basis to uncover how mitochondrial genome involves in various biological processes.

## Supplementary Information

Supplementary Information 1.

Supplementary Information 2.

Supplementary Information 3.

Supplementary Information 4.

Supplementary Information 5.

Supplementary Information 6.

Supplementary Information 7.

Supplementary Information 8.

Supplementary Information 9.

Supplementary Information 10.

Supplementary Information 11.

Supplementary Information 12.

Supplementary Information 13.

## Data Availability

Raw data are available on request.
