# Author Correction: Generation of somatic mitochondrial DNA-replaced cells for mitochondrial dysfunction treatment

**DOI:** 10.1038/s41598-021-99936-z

**Published:** 2021-10-12

**Authors:** Hideki Maeda, Daisuke Kami, Ryotaro Maeda, Akira Shikuma, Satoshi Gojo

**Affiliations:** 1 Department of Cardiovascular Medicine, Kyoto Prefectural University of Medicine, 465 Kajii cho, Kamigyo ku, Kyoto, 602-8566 Japan; 2 Department of Regenerative Medicine, Kyoto Prefectural University of Medicine, 465 Kajii cho, Kamigyo ku, Kyoto, 602-8566 Japan

Correction to: *Scientific Reports* 10.1038/s41598-021-90316-1, published online 25 May 2021

The original version of this Article contained an error in the long term culture of Figure 2(g). As a result,

"ρ(-)".

now reads:

"Mock".

Consequently, Figure 2(g) legend has been modified accordingly,

“MirCs were generated from mitochondrial disease patient-derived (7S) fibroblasts. (**a**) mtDNA CN during the procedure of MirC generation. Fibroblasts that received gene transfer, designated as 7S_ρ(-) were cultivated with or without isolated mitochondria. Mock transfectants that received a plasmid without the endonuclease, designated as 7S_Mock, were subjected to the same protocol. (n = 9, respectively). (**b**) TaqMan qPCR SNP genotyping assay demonstrated the dominance of exogenous mtDNA. MirCs derived from 7S fibroblasts were designated as 7S_MirC. (n = 3, respectively). (**c**) Heteroplasmic sc-ddPCR discriminated three different populations: healthy homoplasmic cells (Cluster 1: CL1, red), heteroplasmic cells (CL2, brown), and mutated homoplasmic cells (CL3, blue) for mtDNA. Representative analyses are shown in the quadrant plotting, and the averages are depicted as a bar graph. Donor mitochondria for MirCs were isolated from EPC100 cells. (n = 3, respectively). (**d**) Cell growth of MirCs compared with the original cells and ρ(-) cells by using time-lapse imaging recorder from day 7 to day 12 in the protocol. The confluency was automatically calculated by JuLI STAT software. (**e**) Microscopic photographs of cell cultures following mitochondrial replacement 5 days after replating at a concentration of 1 × 10^5^ cells on day 12 in the protocol. (**f**) The yield of cells and the doubling time of MirCs were similar to those of 7S fibroblasts. The black bar indicates 200 µm. (n = 3, respectively). (**g**) Long-term culture showed the lifespan extension of MirCs. (n = 3, respectively). (**h**) The cell size of MirCs was maintained during culture, whereas that of the original cells was significantly enlarged from early PDL with time. (n = 3, respectively). (**i**) Short tandem repeats (STRs) demonstrated no contamination of the original MirCs by EPC100 cells that provided the donor mitochondria for MirCs. (**j**) TERT expression in MirCs to deny carcinogenic transformations. The full-length gel of cropped gels is shown in Supplementary Fig. S4. mtDNA, mitochondrial DNA. CNT, no treatment control cell. ρ(-), rho minus, indicates cells with a low mtDNA number. CN, copy number. MC, medium change. DT, doubling time. PDL, population doubling level.”

now reads:

“MirCs were generated from mitochondrial disease patient-derived (7S) fibroblasts. (**a**) mtDNA CN during the procedure of MirC generation. Fibroblasts that received gene transfer, designated as 7S_ρ(-) were cultivated with or without isolated mitochondria. Mock transfectants that received a plasmid without the endonuclease, designated as 7S_Mock, were subjected to the same protocol. (n = 9, respectively). (**b**) TaqMan qPCR SNP genotyping assay demonstrated the dominance of exogenous mtDNA. MirCs derived from 7S fibroblasts were designated as 7S_MirC. (n = 3, respectively). (**c**) Heteroplasmic sc-ddPCR discriminated three different populations: healthy homoplasmic cells (Cluster 1: CL1, red), heteroplasmic cells (CL2, brown), and mutated homoplasmic cells (CL3, blue) for mtDNA. Representative analyses are shown in the quadrant plotting, and the averages are depicted as a bar graph. Donor mitochondria for MirCs were isolated from EPC100 cells. (n = 3, respectively). (**d**) Cell growth of MirCs compared with the original cells and ρ(-) cells by using time-lapse imaging recorder from day 7 to day 12 in the protocol. The confluency was automatically calculated by JuLI STAT software. (**e**) Microscopic photographs of cell cultures following mitochondrial replacement 5 days after replating at a concentration of 1 × 10^5^ cells on day 12 in the protocol. (**f**) The yield of cells and the doubling time of MirCs were similar to those of 7S fibroblasts. The black bar indicates 200 µm. (n = 3, respectively). (**g**) Long-term culture showed the lifespan extension of MirCs. (n = 3, respectively). Mock means cells that received gene transfection procedure without plasmid and subsequently were co-incubated with isolated mitochondria with the same protocol as MirC generation. (**h**) The cell size of MirCs was maintained during culture, whereas that of the original cells was significantly enlarged from early PDL with time. (n = 3, respectively). (**i**) Short tandem repeats (STRs) demonstrated no contamination of the original MirCs by EPC100 cells that provided the donor mitochondria for MirCs. (**j**) TERT expression in MirCs to deny carcinogenic transformations. The full-length gel of cropped gels is shown in Supplementary Fig. S4. mtDNA, mitochondrial DNA. CNT, no treatment control cell. ρ(-), rho minus, indicates cells with a low mtDNA number. CN, copy number. MC, medium change. DT, doubling time. PDL, population doubling level.”

The original Figure 2 and accompanying legend appear below.

The original Article has been corrected.

**Figure 2:**
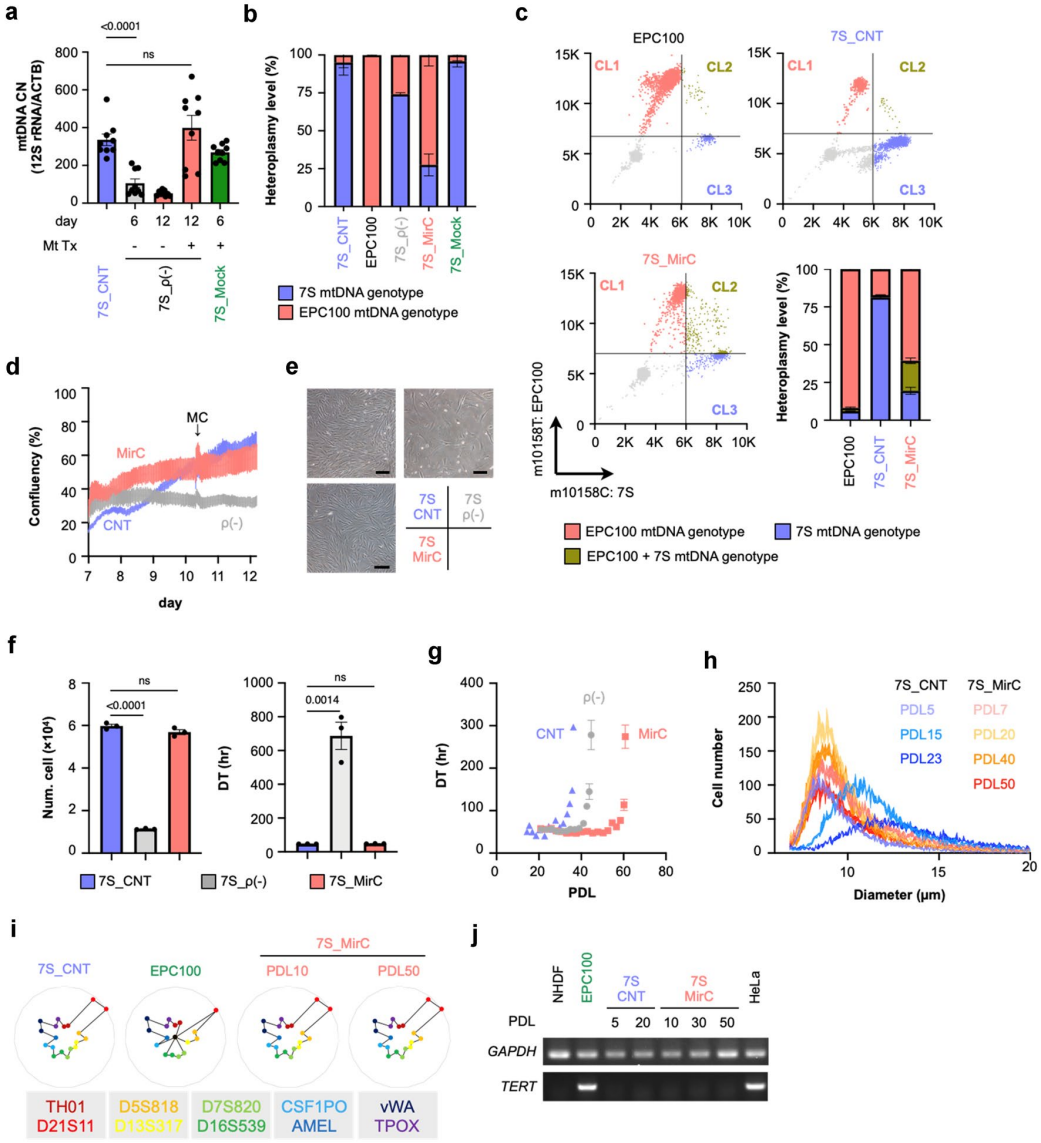
MirCs were generated from mitochondrial disease patient-derived (7S) fibroblasts. (**a**) mtDNA CN during the procedure of MirC generation. Fibroblasts that received gene transfer, designated as 7S_ρ(-) were cultivated with or without isolated mitochondria. Mock transfectants that received a plasmid without the endonuclease, designated as 7S_Mock, were subjected to the same protocol. (n = 9, respectively). (**b**) TaqMan qPCR SNP genotyping assay demonstrated the dominance of exogenous mtDNA. MirCs derived from 7S fibroblasts were designated as 7S_MirC. (n = 3, respectively). (**c**) Heteroplasmic sc-ddPCR discriminated three different populations: healthy homoplasmic cells (Cluster 1: CL1, red), heteroplasmic cells (CL2, brown), and mutated homoplasmic cells (CL3, blue) for mtDNA. Representative analyses are shown in the quadrant plotting, and the averages are depicted as a bar graph. Donor mitochondria for MirCs were isolated from EPC100 cells. (n = 3, respectively). (**d**) Cell growth of MirCs compared with the original cells and ρ(-) cells by using time-lapse imaging recorder from day 7 to day 12 in the protocol. The confluency was automatically calculated by JuLI STAT software. (**e**) Microscopic photographs of cell cultures following mitochondrial replacement 5 days after replating at a concentration of 1 × 10^5^ cells on day 12 in the protocol. (**f**) The yield of cells and the doubling time of MirCs were similar to those of 7S fibroblasts. The black bar indicates 200 µm. (n = 3, respectively). (**g**) Long-term culture showed the lifespan extension of MirCs. (n = 3, respectively). (**h**) The cell size of MirCs was maintained during culture, whereas that of the original cells was significantly enlarged from early PDL with time. (n = 3, respectively). (**i**) Short tandem repeats (STRs) demonstrated no contamination of the original MirCs by EPC100 cells that provided the donor mitochondria for MirCs. (**j**) TERT expression in MirCs to deny carcinogenic transformations. The full-length gel of cropped gels is shown in Supplementary Fig. S4. mtDNA, mitochondrial DNA. CNT, no treatment control cell. ρ(-), rho minus, indicates cells with a low mtDNA number. CN, copy number. MC, medium change. DT, doubling time. PDL, population doubling level.

