# Author Correction: Generation of somatic mitochondrial DNA-replaced cells for mitochondrial dysfunction treatment

**DOI:** 10.1038/s41598-021-03589-x

**Published:** 2021-12-10

**Authors:** Hideki Maeda, Daisuke Kami, Ryotaro Maeda, Akira Shikuma, Satoshi Gojo

**Affiliations:** 1 Department of Cardiovascular Medicine, Kyoto Prefectural University of Medicine, 465 Kajii cho, Kamigyo ku, Kyoto, 602‑8566 Japan; 2 Department of Regenerative Medicine, Kyoto Prefectural University of Medicine, 465 Kajii cho, Kamigyo ku, Kyoto, 602‑8566 Japan

Correction to: *Scientific Reports* 10.1038/s41598-021-90316-1, published online 25 May 2021

The original version of this Article contained an error in the Results, under the subheading ‘Macropinocytosis of exogeneous mitochondria is regulated by the mTORC1 pathway’,

“Essential amino acid (EAA)-free medium for AMPK stimulation, palmitic acid (PA) for mTORC1 activation, and rapamycin for mTORC1 suppression were chosen to examine the state of ρ(-) cells."

now reads:

“Essential amino acid (EAA)-free medium for AMPK stimulation, phosphatidic acid (PA) for mTORC1 activation, and rapamycin for mTORC1 suppression were chosen to examine the state of ρ(-) cells."

The original Article has been corrected.

