# Supplementary material for: Generation of somatic mitochondrial DNA-replaced cells for mitochondrial dysfunction treatment
Source: Sci Rep. 2021 May 25;11:10897. doi: 10.1038/s41598-021-90316-1 (PMC8149667; doi:10.1038/s41598-021-90316-1)

**Supplementary Figures**

**Generation of Somatic Mitochondrial DNA-Replaced Cells for Mitochondrial Dysfunction Treatment**

Hideki Maeda^1+^, Daisuke Kami^2+^, Ryotaro Maeda^1^, Akira Shikuma^1^, Satoshi Gojo^2*^

^1^Department of Cardiovascular Medicine,

^2^Department of Regenerative Medicine,

Kyoto Prefectural University of Medicine

**Contents**

Figure legend for Supplementary Figure S1 to S7 and Movies S1 to S4

Figure legend for Table S1 and S2

**
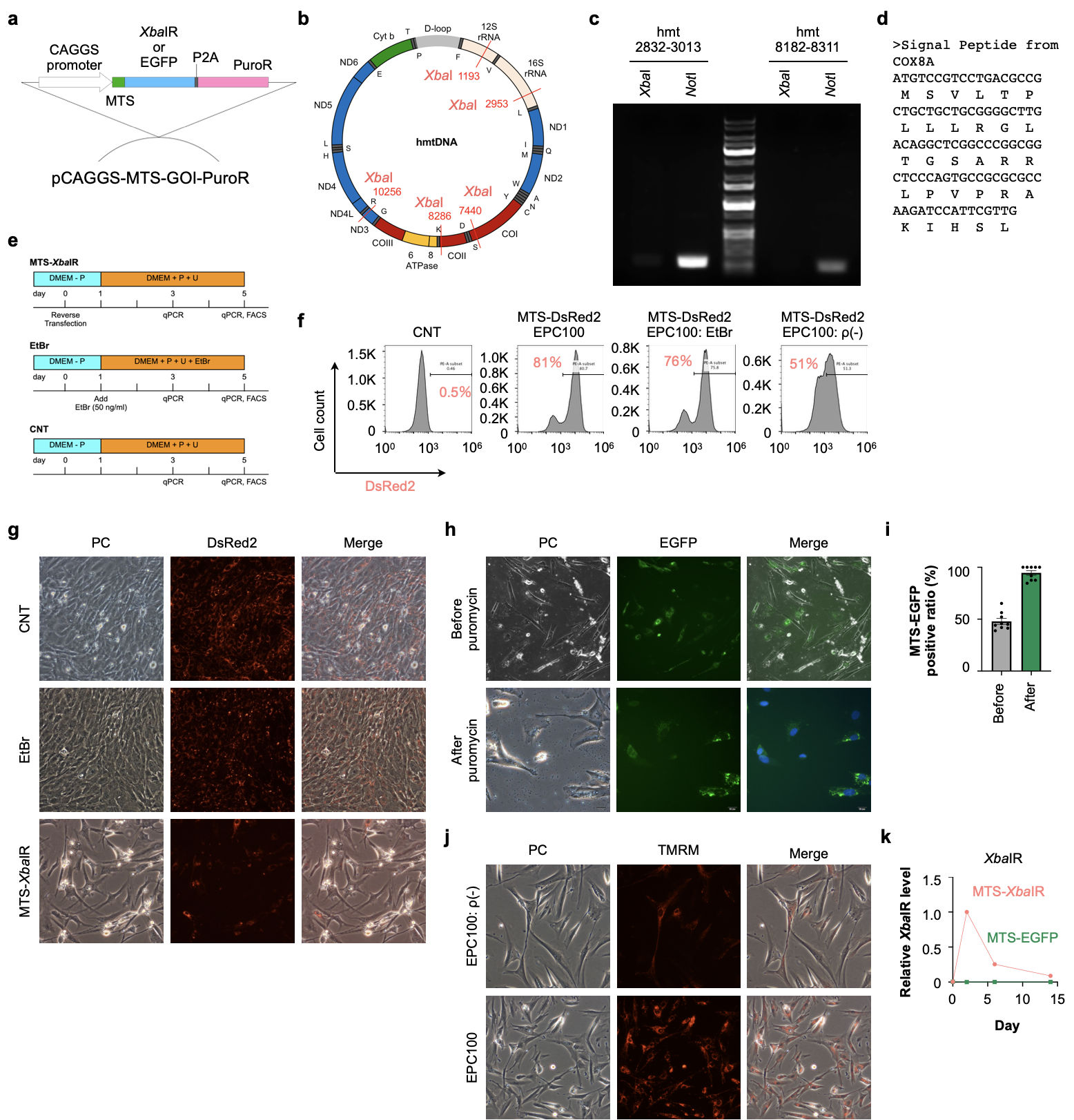
**

**Supplementary Figure S1. The generation of ρ(-) cells by mitochondria-targeted endonuclease.** (a) Map of the plasmid carrying endonuclease XbaIR (GenBank: AF051092.1) or EGFP and mitochondria targeting sequence, which was used to decrease mtDNA levels. (b) Cambridge reference sequence for human mtDNA, indicating the five restriction sites for XbaIR. (c) Electrophoresis following digestion of human mtDNA with an endonuclease, either XbaIR or NotI. (d) Mitochondria-targeting sequence (MTS) of COX8A, pCAGGS-MTS-XbaIR-PuroR. (e) Time schedule used to examine the efficiency of MTS-XbaIR to decrease mtDNA levels compared with a conventional method using ethidium bromide for mtDNA depletion, DMEM: pyruvate-free DMEM (Fujifilm Wako Chemical Corp., 044-29765), P: pyruvate (100 µg/ml), U: uridine (50 µg/ml). (f) By using EPC100, which was labeled with DsRed2 in mitochondria, changes in the intensity of DsRed2 were considered to represent a change in mtDNA content. ρ(-) cells showed a drastic decrease in DsRed2 compared with cells exposed to EtBr in FACS analyses on day 5. (g) Fluorescent images of mtDNA reductions in ρ- cells of genetically modified EPC100 compared with EtBr treatment on day 5. (h) Efficient puromycin selection to generate ρ(-) cells for 24 h. EGFP was used as a marker to successfully detect gene-transferred cells. (i) Puromycin selection enriched cells to 2 folds. (n=3, respectively) (j) Another surrogate fluorescent marker of mitochondrial content, TMRM, showed a clear decrease in EPC100 fluorescence in ρ(-) cells on day 5 of the protocol. (k) Kinetics of EGFP following plasmid transfection showed a sharp peak on day 2 and disappeared until day 14 (n=3). PC; phase contrast


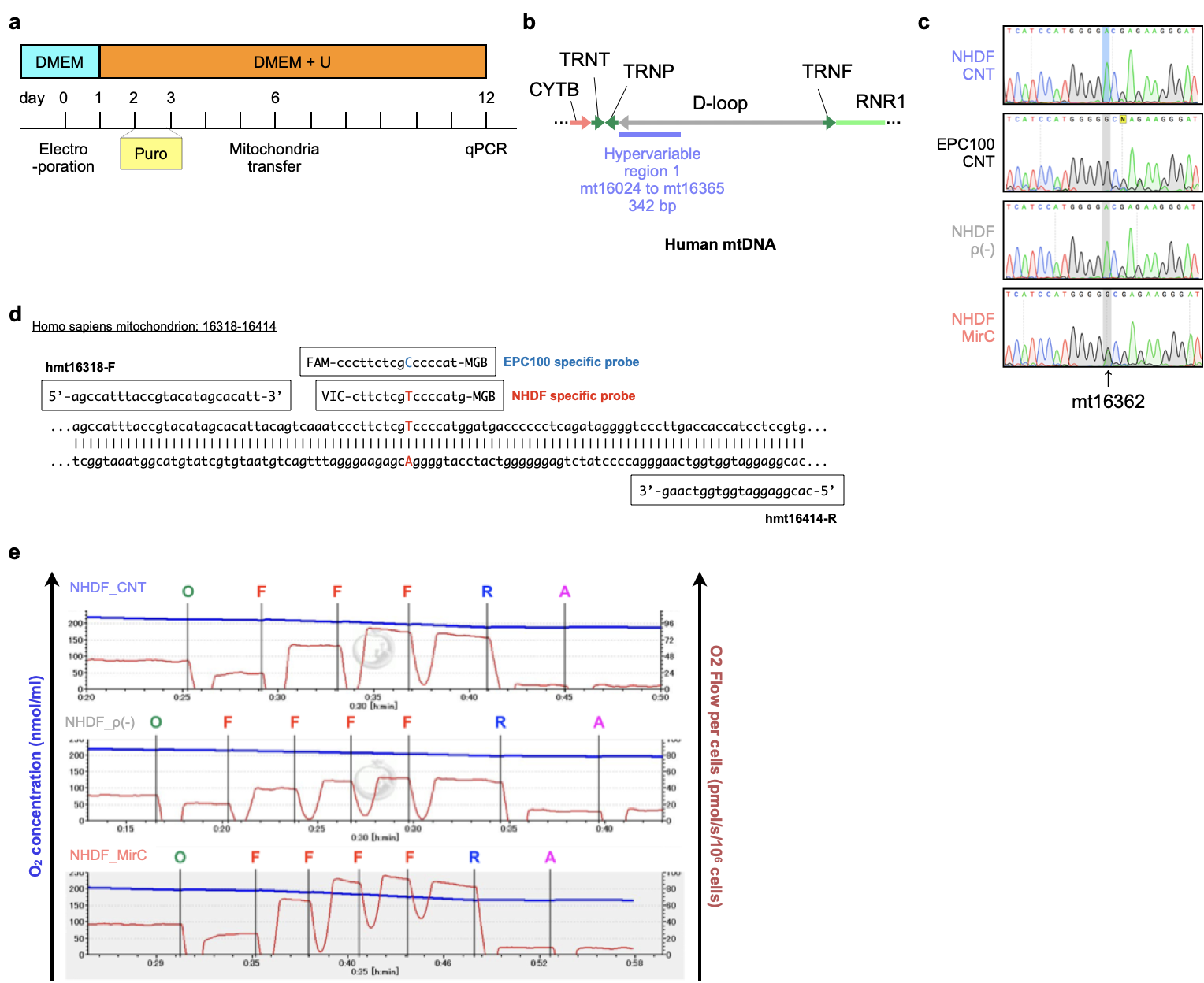


**Supplementary Figure S2. TaqMan SNP genotyping design and respirometry of MirCs**

(a) Protocol of mtDNA replacement, DMEM: pyruvate-free DMEM (Fujifilm Wako Chemical Corp., 044-29765), U: uridine (50 µg/ml). (b) The fragment of human mtDNA in which primer sets amplify the region encompassing a single nucleotide difference in the D-loop between NHDFs and EPC100 mapped on the Cambridge reference sequence. (c) Sequence data for the D-loop in NHDFs and EPC100, showing the difference in the position of hmt16362, where adenine [A] for NHDFs and guanine [G] for EPC100. ρ(-) cells were validated with the same sequence as the parental cells, NHDFs. MirCs derived from NHDFs and isolated mitochondria from EPC100 cells showed chimerism of A and G. (d) The probe and primer sets for detecting a single nucleotide difference. (e) Respirometry showed that MirCs were similar to parental cells, whereas ρ(-) cells were severely damaged. (n=3, respectively)

**
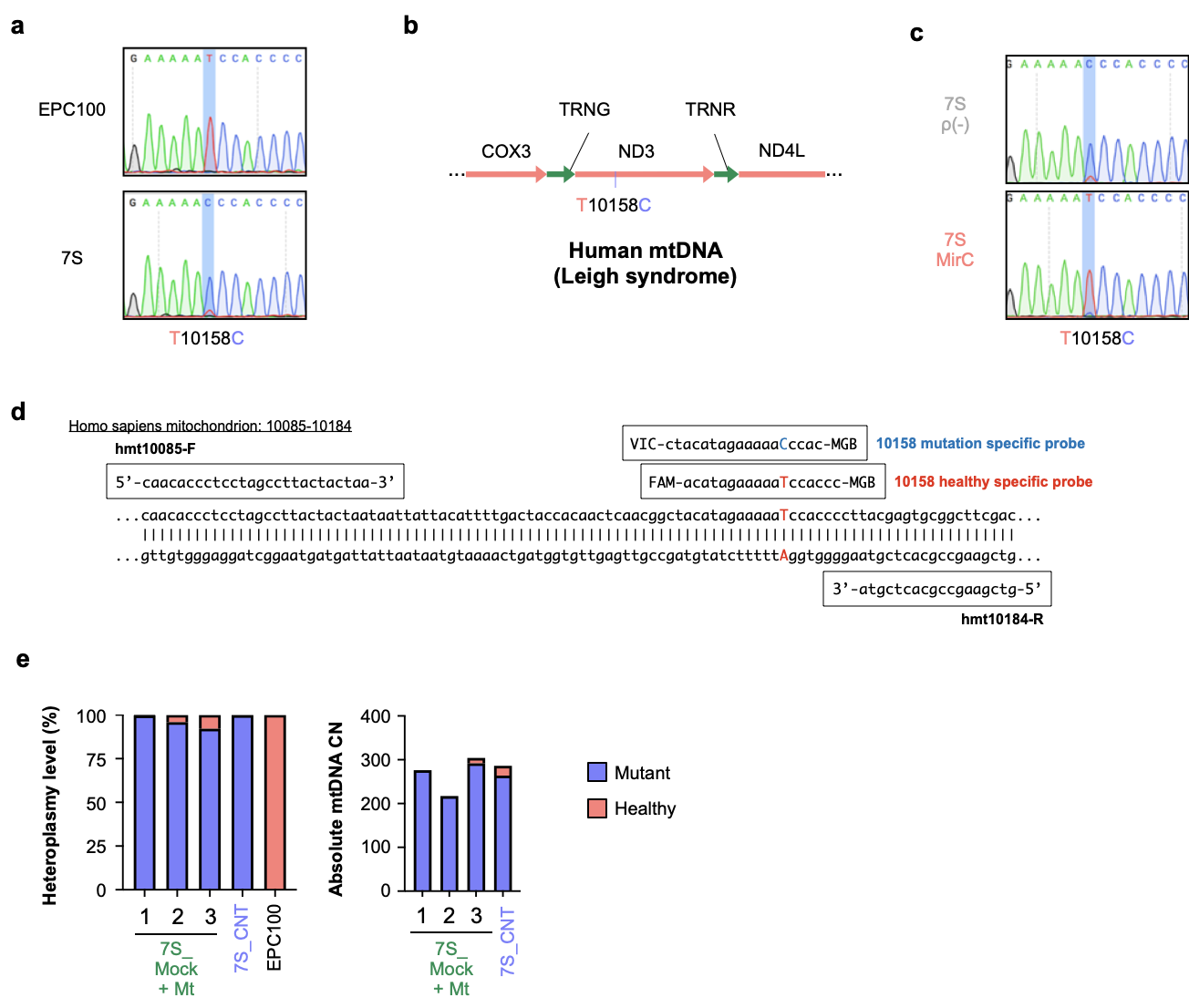
**

**Supplementary Figure S3. Data on mitochondrial disease patient-derived fibroblasts.** (a) Sequence of mtDNA in Leigh syndrome patient-derived fibroblasts, called 7S fibroblasts, showing the hmt10158 T to C mutation and high heteroplasmy of the mutation with a minor portion of T. (b) Primer set for the TaqMan SNP genotyping assay indicated in the Cambridge reference sequence. (c) Sequence in MirCs derived from 7S fibroblasts and mitochondria from EPC100 showed that a major wave was T, which is an exogenous nucleotide. (d) The probe set for the TaqMan SNP assay was mapped on the amplified sequence in ND3 with the primer set. (e) Mock transfection in three independent experiments in 7S fibroblasts did not significantly impact heteroplasmy or mtDNA CN, showing similar levels to the original cells.


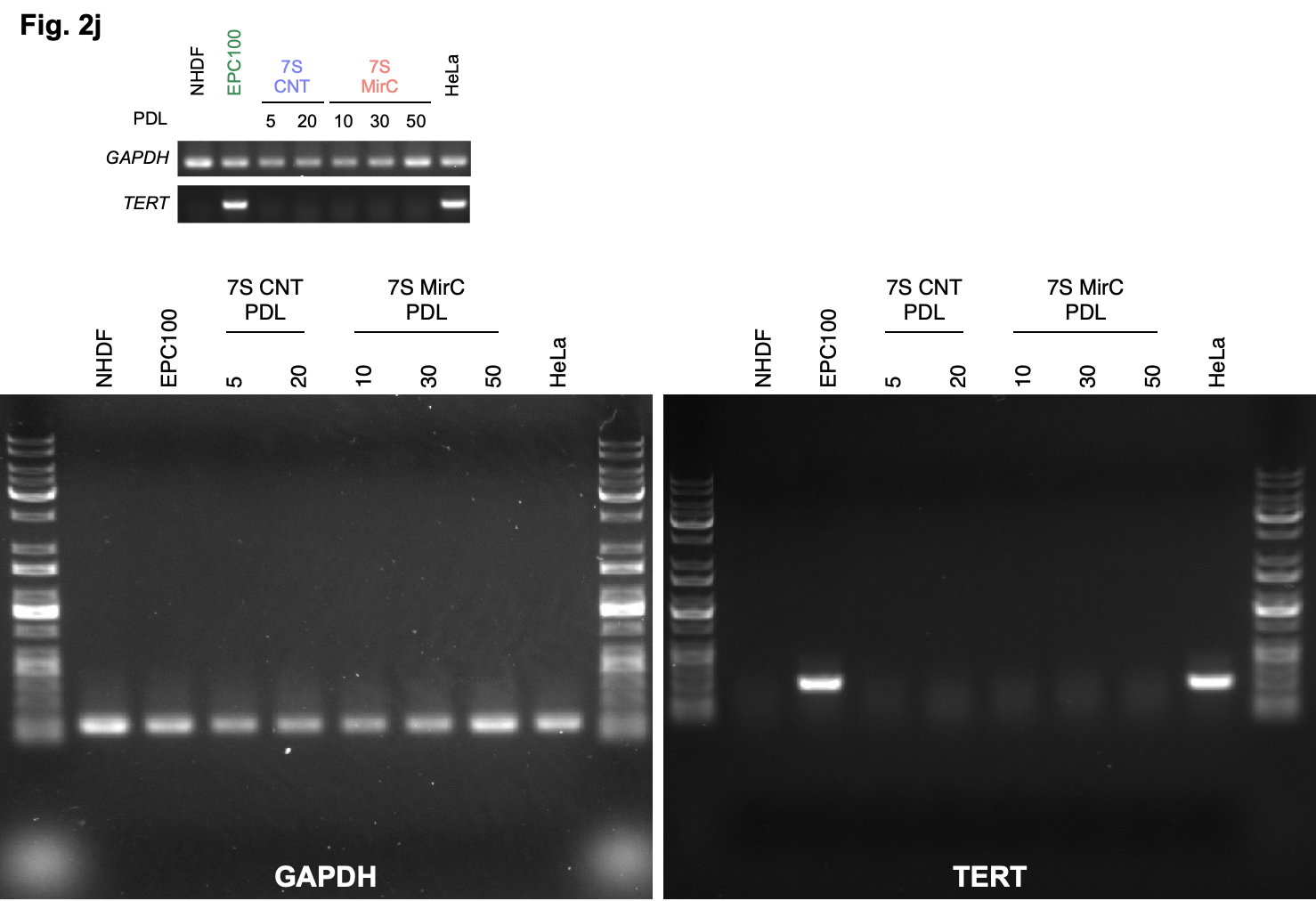


**Supplementary Figure S4. Agarose gel analysis related to Figure 2j.** Agarose gel images show the results of TERT and GAPDH expression.


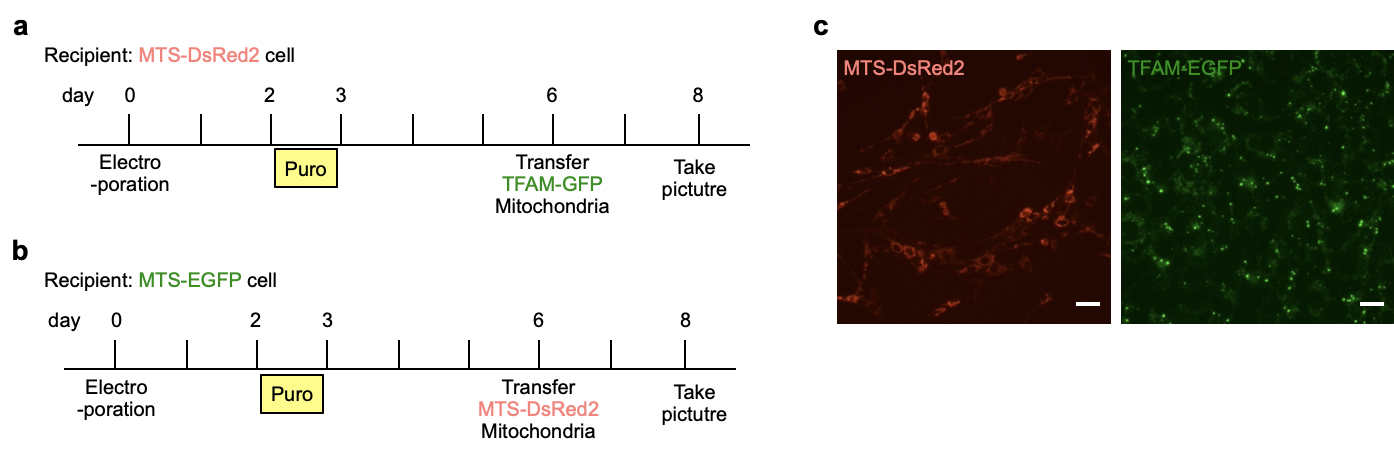


**Supplementary Figure S5. The protocol of mtDNA replacement.** (a) In combination of TFAM-labeled mitochondria with EGFP as the donor and cells with mitochondria with DsRed2 as the recipient, the protocol to generate MirCs was executed to investigate the fate of mitochondrial nucleoids in the donor mitochondria. (b) The protocol of MirC generation along with mitochondria marked with DsRed2 as the donor and cells with genetically marked mitochondria with EGF as the recipient. (c) Fluorescence microscopic images of cells transfected with MTS-DsRed2 or TFAM-EGFP carrying recombinant retrovirus. White bar indicates 50 µm.

**
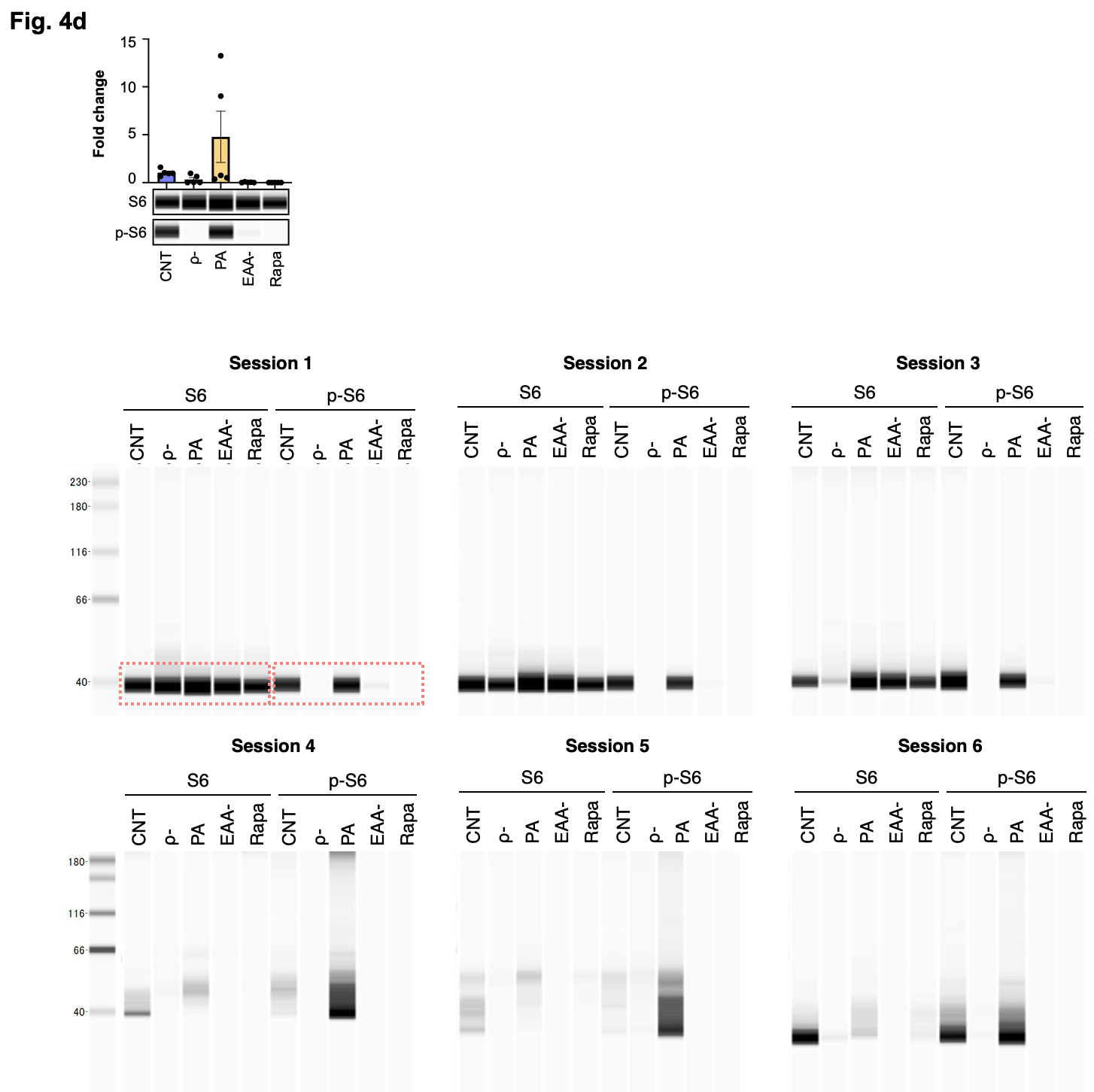
**

**Supplementary Figure S6. Phosphoprotein analysis related to Figure 4d.** Wes capillary data are shown for six individual experiments. The red dotted line is the area used in Figure 4d. CNT, control, Nothing to process; ρ-, cells with *Xba*IR treatment; PA, palmitic acid treatment; EAA-, essential amino acid-free; Rapa, rapamycin treatment.


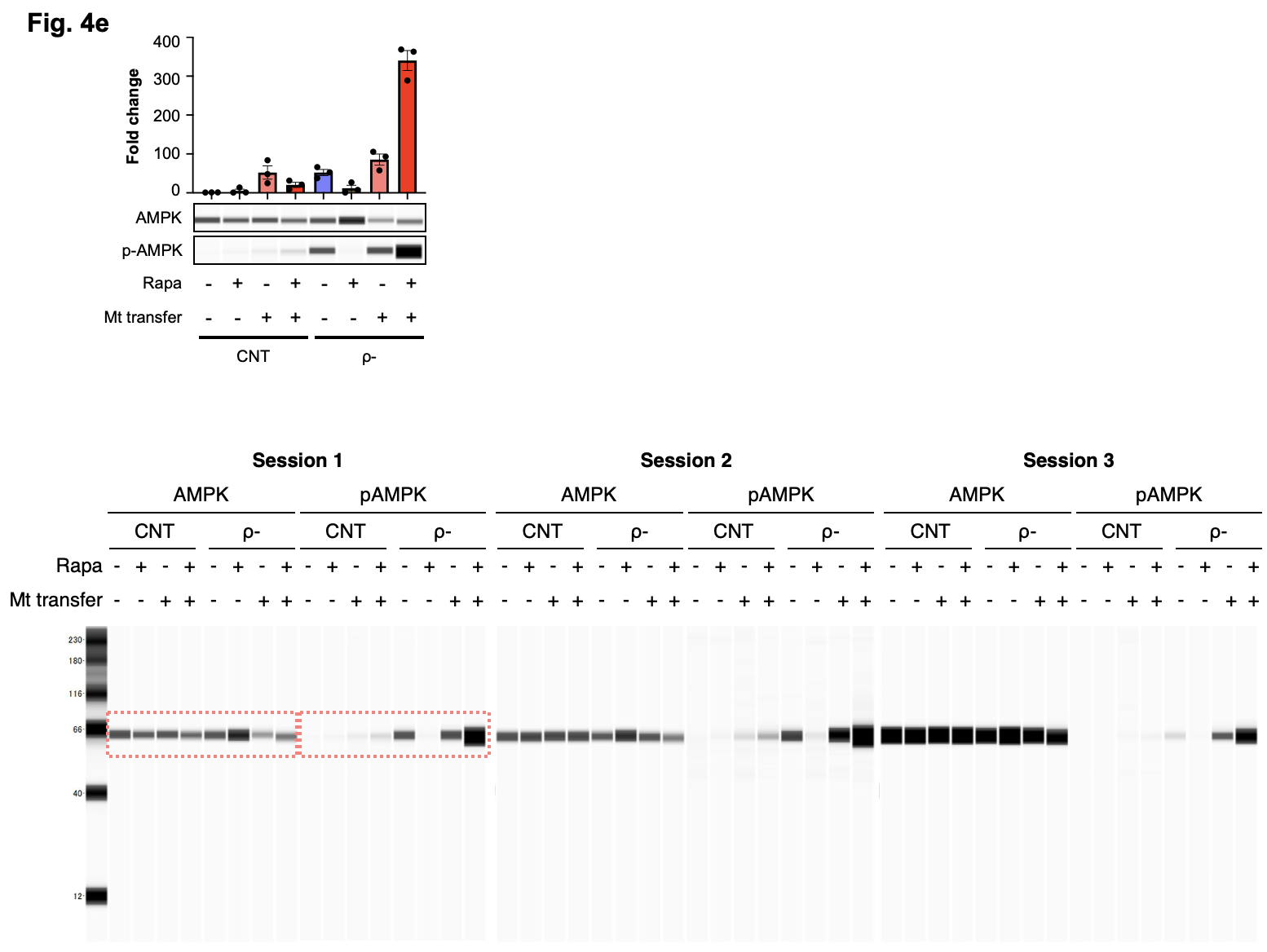


**Supplementary Figure S7. Phosphoprotein analysis related to Figure 4e.** Wes capillary data are shown for three individual experiments. The red dotted line is the area used in Figure 4e. CNT, control, not to process; ρ-, cells with *Xba*IR treatment.

**Movies supplement 1 to supplement 4**

**Supplementary Movie 1.** Direct contact of exogenous mitochondria with endogenous mitochondria was recognized. Green: EGFP-marked mitochondria in the single host cell, red: DsRed2-marked exogenous mitochondria, 4 independent cells (1-1 to 1-4).

**Supplementary Movie 2.** Mitochondrial DNA deletion suppresses exogenous mitochondrial internalization: Exposure of antimycin A and oligomycin (A/O) to PARKIN-overexpressing C2C12 cells efficiently provided ρ0 cells, which were coincubated with exogenous DsRed2-marked mitochondria. 2-1: A/O + with mitochondria, 2-2: A/O – with mitochondria, 2-3: A/O + without mitochondria, 2-4: A/O – without mitochondria

**Supplementary Movie 3.** Time-lapse imaging of NHDFs with DsRed2-marked mitochondria. 3-1: ρ(-) NHDFs with mitochondria, 3-2: Naïve NHDFs with mitochondria, and 3-3: Mock transfectant of NHDFs with mitochondria

**Supplementary Movie 4.** Time-lapse imaging of ρ(-) 7S fibroblasts with DsRed2-marked mitochondria.

**Supplementary Table S1. Sequences of primers and probes**

| **Table S1** | | |
| --- | --- | --- |
| ***PCR primers to DNA*** | | |
| **Gene** | **Forward sequence** | **Reverse sequence** |
| hmt2832-3013 | AACCCAACCTCCGAGCAGTA | CATCGGGATGTCCTGATCCA |
| hmt8182-8311 | CTGTGGAGCAAACCACAGTT | AAGTTAGCTTTACAGTGGGC |
| hmt12S rRNA | GTTTTAGATCACCCCCTCCCC | TCGTAGTGTTCTGGCGAGC |
| hmtDloop | CTCTGTTCTTTCATGGGGAAGC | CATAAACTGTGGGGGGTGTCT |
| hmtHV2 | GGGTATGCACGCGATAGCAT | GAGATGTGTTTAAGTGCTGTGGC |
| hmtND3 | GTCTCCATCTATTGATGAGGGTC | GGATATGAGGTGTGAGCGAT |
| ACTB | CCGTGCTCAGGGCTTCTTGT | GTGCTCGATGGGGTACTTCAG |
| ***qPCR primers to DNA*** |  |  |
| **Gene** | **Forward sequence** | **Reverse sequence** |
| hmt12S rRNA | GTTTTAGATCACCCCCTCCCC | TCGTAGTGTTCTGGCGAGC |
| ACTB | CCGTGCTCAGGGCTTCTTGT | GTGCTCGATGGGGTACTTCAG |
| ***qPCR primers to RNA*** |  |  |
| **Gene** | **Forward sequence** | **Reverse sequence** |
| XbaIR | CAAGGTGATGACGTGTCCCA | GATGCGGAATCCGGAAAAGC |
| EGFP | CCGACCACATGAAGCAGCAC | CTTCAGCTCGATGCGGTTCAC |
| GAPDH | TCCTCTGACTTCAACAGCGA | GGGTCTTACTCCTTGGAGGC |
| hTERT | GCATTGGAATCAGACAGCAC | CCACGACGTAGTCCATGTTC |
| hIL6 | ACCCCCAATAAATATAGGACTGGA | TTCTCTTTCGTTCCCGGTGG |
| hIL8 | TTTTGCCAAGGAGTGCTAAAGA | AACCCTCTGCACCCAGTTTTC |
| hCXCL1 | AGCTTGCCTCAATCCTGCAT | CCTCTGCAGCTGTGTCTCTC |
| hICAM1 | ATGCCCAGACATCTGTGTCC | GGGGTCTCTATGCCCAACAA |
| ***SNP assay*** | | |
| **Name** | **Sequence** | |
| hmt16318-F-primer | AGCCATTTACCGTACATAGCACATT | |
| hmt16414-R-primer | CACGGAGGATGGTGGTCAAG | |
| NHDF-probe | VIC-CTTCTCGtCCCCATG-MGB | |
| EPC100-probe | FAM-CCCTTCTCGcCCCCAT-MGB | |
| hmt10085-F-primer | CAACACCCTCCTAGCCTTACTACTAA | |
| hmt10184-R-primer | GTCGAAGCCGCACTCGTA | |
| hmt10158-mutation-probe | VIC-CTACATAGAAAAAcCCAC-MGB | |
| hmt10158-healthy-probe | FAM-ACATAGAAAAAtCCACCC-MGB | |
|  |  | m: mouse, h: human |
|  |  |  |
| ***Antibody*** |  |  |
| **Name** | **Catalog number** | **Company** |
| Phospho-AMPKα (Thr172) (40H9) Rabbit mAb | 2535 | Cell Signaling Technology, Inc. |
| AMPKα (23A3) Rabbit mAb | 2603 | Cell Signaling Technology, Inc. |
| Phospho-S6 Ribosomal Protein (Ser235/236) Antibody | 2211 | Cell Signaling Technology, Inc. |
| S6 Ribosomal Protein (5G10) Rabbit mAb | 2217 | Cell Signaling Technology, Inc. |
| Anti-SOX2 antibody | ab75485 | Abcam plc. |
| Anti Oct-3/4 (C-10) | SC-5279 | Santa Cruz Biotechnology, Inc. |
| Anti Nanog | RCAB0003P | REPROCELL USA Inc. |
| Anti-Stage-Specific Embryonic Antigen-4 Antibody, clone MC-813-70 | MAB4304 | Merck KGaA |
| Anti-TRA-1-60 Antibody, clone TRA-1-60 | MAB4360 | Merck KGaA |
| Anti-TRA-1-81 Antibody, clone TRA-1-81 | MAB4381 | Merck KGaA |

**Supplementary Table S2. Raw data of STR assay.**


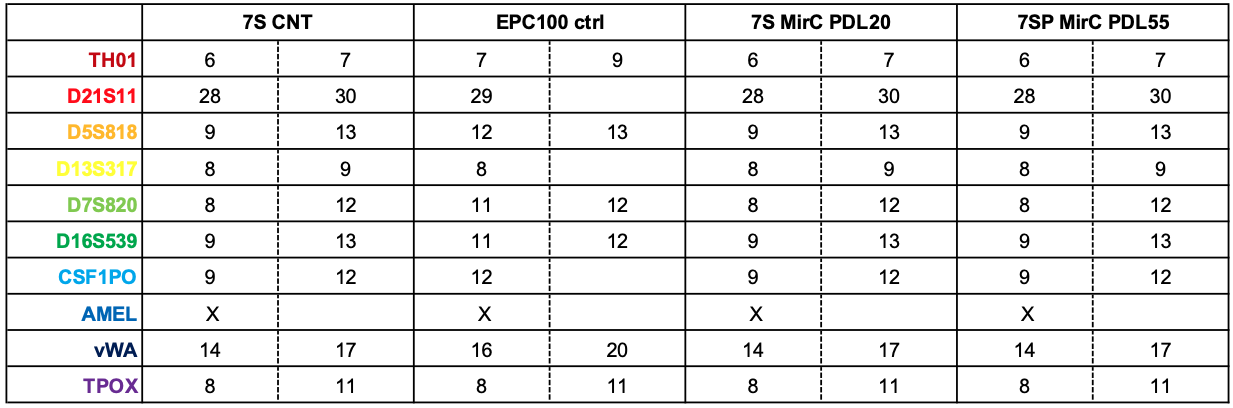

Supplement: Supplementary file 13 — Supplementary Information 13. [file 41598_2021_90316_MOESM13_ESM.docx]
